# The International Caries Classification and Management System (ICCMS™) An Example of a Caries Management Pathway

**DOI:** 10.1186/1472-6831-15-S1-S9

**Published:** 2015-09-15

**Authors:** Amid I Ismail, Nigel B Pitts, Marisol Tellez

**Affiliations:** 1 Maurice H. Kornberg School of Dentistry, Temple University, Philadelphia, 19140, USA; 2 King's College London Dental Institute at Guy's Hospital, King's Health Partners, London, UK

## Introduction

The International Caries Classification and Management System (ICCMS™) is a comprehensive set of clinical protocols that address all diagnostic, preventive and restorative decisions necessary “to preserve tooth structure and restore only when indicated,” which is the mission adopted at the Temple University Caries Management Pathways workshop, in 2012 [1]. The foundation for ICCMS™ is based on extensive critical analyses, research, and clinical feedback on the best approaches to move away from the mechanical or restorative care that has been followed around the world, towards a system where prevention is emphasized, initial caries lesions are prevented from progressing (controlled), and moderate or extensive caries lesions are restored with the goal of preserving, as much as possible, natural tooth structure [2]. This chapter will describe the scientific, and clinical management protocols that have been developed over the last several years by over 70 cariologists, epidemiologists, and clinicians.

While ICCMS™ is recommended as the most comprehensive pathway for caries management to achieve the desired aforementioned mission, it is important to emphasize that ICCMS™ is not the only system available today that promotes staging of the caries process and risk assessment and management [1]. The ICCMS™, in contrast to other systems, has well-developed and documented protocols for the implementation of a new model of caries management. It is based on the well-established and widely used International Caries Detection and Assessment System (ICDAS™).

## What is a caries management pathway?

A caries pathway is a step-by-step protocol or decision map that helps practitioners to intuitively and systematically collect and analyze personal and clinical data to develop comprehensive patient care plans, which are different from the traditional listing of clinical restorative procedures that are usually recommended to a patient. The exclusive focus on restorative care is value-blind because the outcome of the treatment is providing high quality restorations and not the control nor prevention of dental caries. In a comprehensive patient care plan, the focus is on value-based decisions that will enable patients to manage their behavioral practices and risk-tailored preventive care at home or in clinics to prevent and control caries. While restorative care plays a significant role in caries management, it cannot alone control caries.

In some US dental practices, dentists make decisions intuitively [3]. The mental maps that dentists develop, after years of education and practice, are usually made without delving too much into the rationale for each decision step. A management (or practice) pathway by contrast is a systematic guide that enables dentists to follow critical steps to reach a decision or develop a management plan [4].

**Figure 1:**
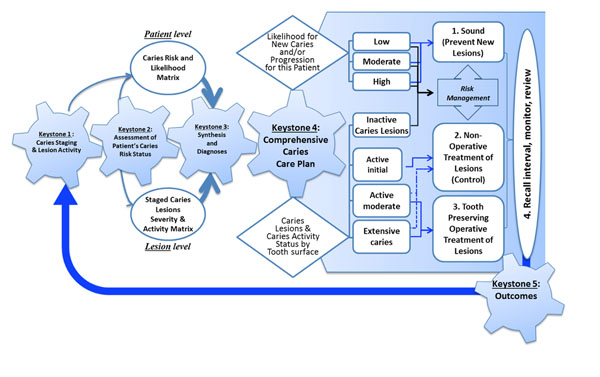
Keystones of the International Caries Classification and Management System (ICCMS™)

The management pathway upon which the ICCMS™ was developed is depicted in Figure 1. The proposed ICCMS™ Comprehensive Patent Care Pathway represents significant progress from the traditional system of treatment planning (or comprehensive treatment planning) that is practiced around the world. Treatment planning focuses on addressing all urgent, surgical, periodontal, restorative and prosthetic care need of patients in a sequenced approach based on urgency of care and the need to build healthy periodontal tissue before proceeding with restorative or prosthetic care.

Comprehensive patient care expands on the system of treatment planning to include; prevention of early and advanced disease, preservative management of diseased tissues, behavioral change of patients to enable them to promote their own health and achieve health outcomes that are based on preservation of tooth and periodontal issues, and oral mucosal health. Comprehensive patient care should synthesize and integrate all contemporary knowledge of the biological, social, behavioral, cultural, systemic, and dental/oral risk factors associated with disease development and progression of oral and dental diseases and conditions. Comprehensive oral health care enables patients from the first dental visit to play a pivotal role in their own oral health as well as providing clinical care.

Caries management models have been debated for some time, and international workshops have been held during the last several years to define pathways for comprehensive caries care. The ICCMS™is a pathway for comprehensive patient care that fits well with the concepts agreed in the policy on caries classification and management set out by the FDI Word Dental Federation in 2013 [5].

Building on serial developments in Caries Classification from 2002 by the ICDAS Foundation, and on Caries Management Meetings held in 2010 and 2011, an international workshop was held at Temple University Maurice H. Kornberg School of Dentistry in 2012 to review different systems for caries detection, risk assessment, and caries management [1,6-8]. While several systems were reviewed during that workshop, no common pathway was agreed upon. A consensus, however, was reached among the experts in the field of caries research and management that the common mission of any new caries management pathway should be to

Preserve tooth structure and restore^*^ only when indicated

* Surgical removal of hard dental tissues

The workshop participants also defined the following goals for a new comprehensive patient care model:

1. Achieve and maintain dental health, prevent progression of existing initial lesions and restore moderate or extensive lesions by use of risk-adjusted clinical decision making.

2. Minimize the use of surgical intervention.

The conclusion of that meeting was a review of four caries management systems including the International Caries Classification and Management System (ICCMS™). The developers of the systems were asked to detail how they would achieve the mission and goals. Based on that review and following further workshops in London and Liverpool, the ICCMS™ protocol was written to be a full comprehensive care protocol ready for implementation in dental educational and practice. This chapter here will describe each step of the caries management pathway, which was defined by ICCMS™ using the five important key steps depicted in Figure 1.

## The ICCMS™ caries management pathway

There are five key foundation components (or keystones) of the ICCMS™ pathway that will be explained in this chapter. They are 1) the staging of the caries process, 2) caries risk classification, 3) the ICCMS™ decision matrices, 4) ICCMS™ comprehensive patient management plan, and 5) Outcomes of caries management using ICCMS™.

## Keystone #1: Staging of caries lesions

## Clinical staging of coronal caries

For pits and fissures, the caries categories are described as follows (Appendix I. Photographs and radiographs of the lesions described here are available upon request from Dr. Amid I. Ismail):

**Sound surfaces** have no visible caries when viewed clean and dry (ICDAS™ (International Caries Detection and Assessment System (ICDAS™) code 0) [9,10]. Non-carious white or brown marks on tooth surfaces must be differentiated from early caries lesions. (Please refer to Appendix at the end of the chapter for definitions of the ICDAS™ codes).

**Initial stage caries** is characterized by the first visual change in enamel (seen only after prolonged air drying or restricted to the confines of a pit or fissure) (ICDAS™ code 1) **or** a distinct visual change in enamel (seen on a wet or dry surface) that is within or wider than the confines of a pit or fissure (ICDAS™ codes 1 or 2).

**Moderate stage caries** is characterized visually by either localized enamel breakdown (without visual signs of dentinal exposure) (ICDAS™ code 3) or an underlying dark shadow from dentin (ICDAS™ code 4). Enamel breakdown (ICDAS™ code 3) is often viewed best when the tooth is air dried whilst shadowing from dentinal caries (ICDAS™ code 4) is often best seen with the tooth surface wet.

**Extensive stage caries** is characterized by distinct cavitation exposing visible dentin. (ICDAS™ code 5 lesions exhibit cavitation involving less than half the tooth surface and ICDAS™ code 6 involves half of the tooth surface or more).

## For approximal tooth surfaces, the caries categories are described as follows (Appendix)

**Sound surfaces** have no visible caries when viewed clean and dry (ICDAS™ code 0) (Appendix). Non-carious white or brown marks on tooth surfaces must be differentiated from early caries lesions.

**Initial stage caries** is characterized by the first visual change in enamel (seen only after prolonged air drying) (ICDAS™ code 1) or a distinct visual change in enamel (seen on a wet or dry surface) (ICDAS™ code 2). In occulsal surfaces, these lesions appear light or dark brown non-cavitated areas confined to the pits to fissures. On smooth surfaces, these lesions appear as non-cavitated white demineralization bands that parallel the gingival margin.

**Moderate stage caries** is characterized visually by either localized enamel breakdown (without visual signs of dentinal exposure) (ICDAS™ code 3) or an underlying dark shadow from dentin (ICDAS™ code 4). These lesions are usually seen directly from the lingual or buccal directions and where there is a shadow this can be viewed as discolored dentin visible through an apparently intact marginal ridge from the occlusal direction. Enamel breakdown (ICDAS™ code 3) is often viewed best when the tooth is air dried, whilst shadowing from dentinal caries (ICDAS™ code 4) is often best seen with the tooth surface wet.

**Extensive stage caries** is characterized by distinct cavitation exposing visible dentine. (ICDAS™ code 5 lesions exhibit cavitation involving less than half the tooth surface and ICDAS™ code 6 lesions involve at least half of a tooth surface).

## For buccal-lingual smooth surfaces, the caries categories are described as follows

Sound surfaces have no visible caries when viewed clean and dry (ICDAS™ code 0). Developmental defects like enamel hypoplasias; fluorosis; tooth wear (attrition, abrasion and erosion), and extrinsic or intrinsic stains should be recorded as sound in the absence of other signs of caries lesions.

**Initial stage caries** is characterized by the first visual change in enamel (seen only after prolonged air drying) (ICDAS™ code 1) or a distinct visual change in enamel (seen on a wet or dry surface) (ICDAS™ code and 2). Initial active stage lesions on free smooth surfaces are located in close proximity (in touch or within 1 mm) to the gingival margin or adjacent to orthodontic or prosthetic attachments on a tooth surface.

**Moderate stage caries** is characterized visually by either localized enamel breakdown (without visual signs of dentinal exposure) (ICDAS™ code 3) or an underlying dark shadow from dentin (ICDAS™ code 4). Enamel breakdown (ICDAS™ code 3) is often viewed best when the tooth is air dried, whilst shadowing from dentinal caries (ICDAS™ code 4) is often best seen with the tooth surface wet.

**Extensive stage caries** is characterized by distinct cavitation exposing visible dentin. (ICDAS™ code 5 lesions exhibit cavitation involving less than half the tooth surface and ICDAS™ code 6 involves half of the tooth surface or more).

Interested readers can find detailed explanations of all of the ICDAS™codes in Additional file S1 with clinical examples provided in additional file 2.

## Coronal caries lesion activity criteria

In addition to caries lesion staging, the ICCMS™ pathway requires assessment of the activity state of each lesion. Unfortunately, there are no current valid biological nor clinical tools to assess caries activity; hence, clinicians should rely on clinical indicators, which are defined as follows:

An **Active Lesion** is considered to have a greater likelihood of transition (progress, arrest or regress) than an inactive lesion.

An **Inactive (arrested) Lesion** is considered to have a lesser likelihood of transition than an active lesion.

Clinical observations to be taken into consideration for assessing enamel lesion activity are based on the modifications of the Nyvad et al.[11,12] and the Ekstrand et al.[13] caries lesion activity assessment criteria and include visual appearance, tactile feeling, potential for plaque accumulation and for lesions located near the gingiva - gingival status. The characteristics of active coronal lesions (not all should be present to decide on activity status) are described in Table 1.

**Table 1:** Characteristics of Active and Inactive Caries Lesions

ICDAS Code	Characteristics of Lesion	
	**Signs of Active Lesions**	**Signs of Inactive Lesions**

ICDAS Initial and moderate Stage	Surface of enamel is whitish/yellowish; opaque with loss of luster; feels rough when the tip of the probe is moved gently across the surface. Lesion is in a plaque stagnation area, i.e. in the entrance of pits and fissures, or near the gingival, and in approximal surfaces below or above the contact point. The lesion may be covered by thick plaque prior to cleaning.	Surface of enamel is whitish, brownish or black. Enamel may be shiny and feels hard and smooth when the tip of the probe is moved gently across the surface. For smooth surfaces, the caries lesion is typically located at some distance from the gingival margin. Lesion was not covered by thick plaque prior to cleaning.

ICDAS Extensive Stage	Dentin feels soft or leathery on probing.	Dentin is shiny and hard on probing.

## Radiographic staging of coronal caries lesion

Radiographic information adds significantly to clinical findings in terms of finding lesions at different stages of progression [14-17]. Radiographs help to estimate the depth of caries demineralization into enamel and dentin. Depth is not always associated with the presence of cavitation, especially on approximal surfaces. Clinical investigations in a country with low caries progression rates revealed that, on average, 32% of radiographically visible lesions that extended into the outer third of the dentin manifested cavitation; in contrast, 72% of lesions extending into the inner 2/3 of the dentin were cavitated [18]. Clinically cavitated lesions or lesions with an obvious dentin radiolucency (deeper than the outer 1/3) on the occlusal surface are heavily infected in the dentin beneath the enamel dentin junction [19,20].

Normally two radiographs with time lapse between are required for establish whether the lesion has progressed or not.

The ICCMS™ classifies tooth surfaces radiographically as follows:(modified from . Agustsdottir et al. [21]; Ekstrand et al. [22]) (Refer to the Appendix):

## 0 = No radiolucency

## RA: Initial stages

1= radiolucency in the outer ½ of the enamel

2= radiolucency in the inner ½ of the enamel ± EDJ (enamel-dentin junction) (this stage is recommended for restorative care in the US, if the radiolucency touches the EDJ)

3= radiolucency limited to the outer 1/3 of dentin (in some countries, especially the US, this stage is classified as moderate)

## RB: Moderate stages

4= radiolucency reaching the middle 1/3 of dentin

## RC: Extensive stages

5= radiolucency reaching the inner 1/3 of dentin, clinically cavitated

6= radiolucency into the pulp, clinically cavitated

The reproducibility of this scoring system has been reported to be substantial^17^ or excellent [22]. The accuracy when related to histology using Spearman correlation coefficients was excellent [22].

The evidence indicates that the radiologic penetration depth, at which one can reliably predict the tooth surface is cavitated and the enamel or dentin is heavily infected, is in the region of radiolucency in the middle third of the dentin and deeper. This corresponds to scores 4, 5 and 6 in the ICCMS™ radiographical scoring system. With faster caries progression rates, cavity formation can also be expected in cases scored as 3 in the above system.

## Root Caries Staging and Activity Assessment (Figure 2)

**Figure 2:**
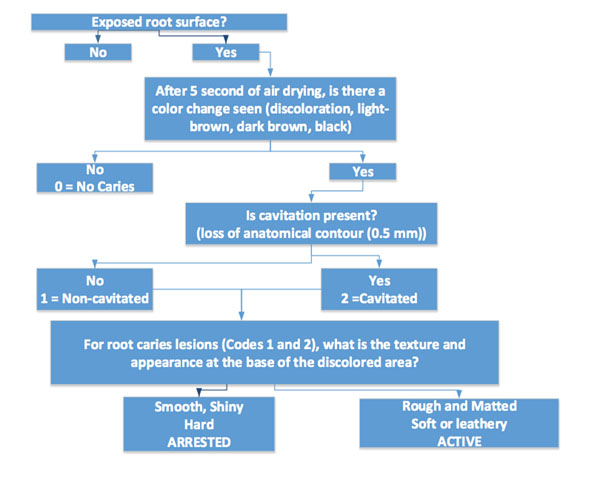
Root Caries Staging and Activity Analysis

ICCMS™ also stages root caries lesions. The staging of root caries is based in assessment of presence of cavitation and activity of the lesions. Again, the emphasis is on prevention, control, and conservative management of lesions.

Root surfaces are classified as follows:

## Code 0

The root surface does not exhibit any unusual discoloration that distinguishes it from the surrounding or adjacent root areas, nor does it exhibit a surface defect either at the cemento-enamel junction or wholly on the root surface. The root surface has a natural anatomical contour, or

The root surface may exhibit a definite loss of surface continuity or anatomical contour that is not consistent with the dental caries process. This loss of surface integrity usually is associated with dietary influences or habits such as abrasion or erosion. These conditions usually occur on the facial (labial) surface. These areas typically are smooth, shiny and hard. Abrasion is characterized by a clearly defined outline with a sharp border, whereas erosion has a more diffuse border. Neither condition shows discoloration.

## Code 1 (initial)

There is a clearly demarcated area on the root surface or at the cemento-enamel junction (CEJ) that is discolored (light/dark brown, black) but there is no cavitation (loss of anatomical contour < 0.5 mm) present.

## Code 2 (Moderate/Extensive lesion)

There is a clearly demarcated area on the root surface or at the cemento-enamel junction (CEJ) that is discolored (light/dark brown, black) and there is cavitation (loss of anatomical contour < 0.5 mm-2 mm (Moderate), > 2mm (Extensive)) present.

## Root caries activity

The characteristics of the base of the discolored area on the root surface can be used to determine whether or not the root caries lesion is active or not. These characteristics include texture (smooth, rough), appearance (shiny or glossy, matte or non-glossy), location in a plaque-stagnation area, and perception of texture on gentle probing (soft, leathery, hard). Active root caries lesions are usually located within 1 mm of the crest of the gingival margin

## Keystone #2: Risk assessment and classification

**Figure 3:**
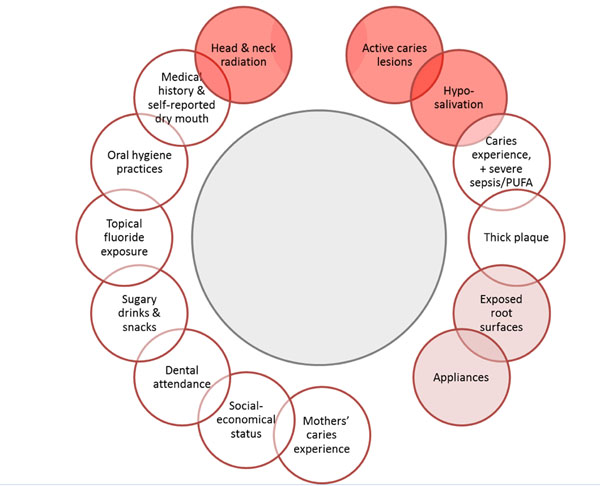
**Caries Risk Factors** PUFA stands for exposed Pulp, Ulceration associated with retained root fragments or sharp edges caused by carious destruction, Fistula, and Abscess Red circles indicate risk factors that classify an individual with high caries risk

The second keystone in caries management is the assessment of caries risk. While this is an important step for caries control and for achieving the overall outcomes of patient care in ICCMS™, the evidence on the predictive validity of current assessment tools is weak[7]. The ICCMS™ requires classifying individuals into low, medium, or high risk categories based on an overall assessment of the risk factors outlined in Figure 3.

The ICCMS™recommends assessing the following risk factors using both interview data and clinical assessment:

**Medical health:** Current use of medications, recreational drugs, or systemic conditions that may cause hyposalivation.

**Head and neck radiation**: Patients undergoing radiotherapy for head and neck cancer are categorized as high risk for developing caries because of the side effects or sequelae of the treatment regimes [23,24]. The symptoms include (but are not limited to) xerostomia or hyposalivation, mucositis (affecting eating and oral hygiene practices) and altered taste sensation (which may result in patients utilizing inappropriate or cariogenic means of addressing the issue).

**Sugary drinks and snack**s: Based on the evidence from systematic reviews and several well-conducted cohort studies [25-27], it can be concluded that there is a significant association, though weaker than in the past under modern dietary practices and fluoride exposure, between higher risk of dental caries and high exposure to sugared beverages and snacks. Therefore, consumption of sugared beverages and snacks needs to be included as a part of a patient's caries risk assessment.

**Low fluoride exposure:** Patients under certain conditions can be considered to have inadequate fluoride exposure if they have the following profiles [28-32]

• No daily use of fluoridated toothpaste (less than 2x daily)

• For children: tooth brushing with non-fluoridated toothpaste

• Concentration of fluoridated toothpaste less than 1000 ppm of fluoride

**Mother's or caregivers’ caries experience:** It is well accepted that development of early childhood caries is influenced by environmental factors beyond individual-level factors, including a mother's or caregiver's dental health. Several mother-child dyad studies reported that there is a significant correlation between mothers’ and children's caries status [33-37], thus suggesting a mother's (or caregiver's) caries status can be a predictor for child's caries development.

**Oral hygiene behaviors:** Poor oral hygiene status as evidenced by accumulated plaque on the dentition can be predictive of caries development and, hence, is a useful risk indicator. However, the relationship between presence of plaque and caries risk is complex because it depends on the presence of cariogenic bacterial species, which is the determinant factor, and, hence, its use in clinical risk assessment must be viewed with caution. A longitudinal study [38] demonstrated that visible plaque on the labial surface of incisors in young children (19 months) was good predictor of caries development at 36 months of follow-up, demonstrating a sensitivity of 83% and specificity of 92%. The research team was able to correctly classify 91% of children with regard to future caries risk using plaque accumulation alone. Wendt et al.[39] also demonstrated that oral hygiene (i.e., toothbrushing) status in infants and toddlers was associated with lower caries risk. In a 7-year follow up study, Tagliaferro et al.[40] found that oral hygiene status was predictive of high caries at baseline but was not predictive of new caries incidence over the 7 year of the study. Mascarenhas[41] reported oral hygiene status to be an important risk indicator for both enamel and dentinal caries in 12 year olds and Mathiesen et al. [42] reported similar results for 14 year olds when brushing with a fluoride dentifrice. Similarly in adults Domejean et al.[43] found that visible heavy plaque increased risk for future caries development (OR 2.55; 2.35-2.76). In assessing oral hygiene practices, ICCMS™ recommends evaluating the frequency and time spent during tooth brushing and flossing, and timing (after meals, before bedtime).

**Socioeconomic status (SES):** Even though definitions of SES may vary, an individual's SES is likely to be an important predictor of caries risk [44,45]. However, the correlation between SES and dental caries has not always been negative. Data from several emerging economies or rich-developing countries show that caries is more prevalent in higher income groups. The same correlation existed in developed countries in the later part of the 19^th^ and early part of the 20^th^ centuries.

Current evidence from the literature indicating a reverse relationship between one's SES and caries level, is mainly based on studies conducted in developed or industrialized countries. Therefore, the relationship might not be applicable to countries at different stages of development. In low-income developing countries, the SES-caries relationship might not be as clear, or can even be reversed (high SES individuals have a higher level of dental caries). Data from the Global Oral Health Data Bank, maintained by the World Health Organization (WHO), suggested that developing countries, where caries prevalence was low initially, experience a high level of caries prevalence as they are industrialized and exposed to refined ‘cariogenic’ foods [46].

The ICCMS™recommends assessing the following risk factors during the clinical examination of patients:

**Active caries lesions** as defined in a previous section.

**Caries experience:** Increased caries risk is associated with the presence of restorations (or extractions) [47-49]. The level of evidence supporting the causal relationship between presence of restorations and increased caries risk is based on a small number of case-control or cohort studies.

There is evidence that the presence of marginal ditching places teeth at increased caries risk [50,51]. The extent of marginal deficiencies will range from those barely perceptible on visual examination alone to those that will readily admit a ball-ended probe. Since an increased width of the marginal deficiency may be a risk factor for the likelihood of developing caries it may be important to have a threshold at which the deficiency is recorded as present or absent. The ICCMS™ recommends that if a ball-ended probe is part of the examination kit, two categories of ditching could be recorded according to whether or not the probe can full enter into the gap between tooth and restoration.

**Thick and undisturbed biofilm:** Dental caries is now considered an endogenous infection caused by a change in the oral microbial ecology (microbiome) resulting in the selection of bacterial species that have the potential to ferment sugars and starch [52,53]. The individual differences and complexity of the microbiome are influenced by transmission of bacterial species between infants and their caregivers as well as other environmental sources including foods, drinks, and all human contacts [54]. Recent evidence indicates dental caries and periodontal diseases occur because of a shift in the microbial ecology and the reduction in bacterial diversity of the microbiome in the oral cavity [55]. The level of evidence supporting the causal relationship between accumulation of a thick layer of biofilm or in stagnation areas and increased caries risk is based on several case control and cross-sectional studies [13,56-62].

**Dry mouth:** There is an increased caries risk associated with xerostomia/hyposalivation. The level of evidence supporting the causal relationship between xerostomia/hyposalivation and increased caries risk is based a small number of case-control or cohort studies [63,64].

**Exposed root surfaces**: Increased risk of root caries is associated with the number of exposed root surfaces. The level of evidence supporting the causal relationship between root caries and exposed root surfaces is supported by one systematic review, and a small number of case-control or cohort studies [65-69].

**Appliances** that may increase development of the biofilm: Increased caries risk is associated with the use of an oral appliance including partial dentures. The level of evidence supporting the causal relationship between use of the oral appliances and increased caries risk is based on a small number of case-control or cohort studies, as well as expert opinions [70-76].

## PUFA (Exposed Pulp, Ulceration associated with retained root fragments or sharp edges caused by carious destruction, Fistula, and Abscess)

Increased caries risk is associated with a higher PUFA score. The level of evidence supporting the causal relationship between an increasing PUFA score and increased caries risk is based on only one study [77].

## Classification of individuals into low, medium, and high risk categories

While the ICCMS™ does not recommend a single tool for assessing caries risk, there is recent evidence confirming that the CARIOGRAM algorithm has good validity in predicting caries development in children [78]. The ICCMS™ integrates the Caries Management by Risk Assessment (CAMBRA) philosophy [79]. The difference between ICCMS™ and the other implementations of CAMBRA is the detailed description of the caries management pathways and global reach of the ICCMS™.

The ICCMS™ caries risk assessment system relies on the clinical synthesis of the examining dentist to classify patients into low, medium or high risk categories. In Figure 3, the presence of active caries lesion, or previous caries experience, PUFA, or signs of xerostomia or dry mouth, or current or previous head and neck radiation, places an individual in the high risk category. The ICCMS™ risk assessment system can be applied using any of the available risk assessment questionnaires; additionally dentists may use their clinical intuition and synthesis skills to classify patients into low, medium or high risk status using the information provided in this paper.

The ICCMS™ considers the presence of any active caries lesion to be an indicator of risk. Individuals are classified as follows:

• No initial, moderate or extensive **active** lesions

• Initial **active** caries lesions only

• Moderate or extensive **active** caries lesions

Risk status is also defined based on the status of the other factors, as follows:

• **Low:** No exposure to high risk factors (Figure 3: red circles), and low level of other risk factors (sugary snacks, oral hygiene practice, fluoride exposure)

• **Moderate:** a stage between low and high caries risk and when the examining dentist cannot rule out that the individual is at low risk of developing caries.

• **High**: Presence of any of the high risk factors in Figure 3 or previous restorations, appliances, heavy accumulation of biofilm, low fluoride exposure, low socioeconomic status, and mothers or caregivers with high caries experience.

## Keystone #3: Decision Matrices for Diagnosis

ICCMS™has two key decision matrices to assist dentists to develop comprehensive patient care plans to manage caries risk status and provide preventive and restorative care. Using the information on active caries lesions, and the patient's risk status, a decision is made on the likelihood of developing future caries activity (Table 2). The matrix integrates the three actives caries lesions classifications (none, initial, moderate/extensive) and the risk-status classification (low, moderate, and high) into a likelihood matrix that classifies individuals into low, moderate, or high likelihood of developing new caries lesions. There are three likelihood categories (low, medium and high). For each of these categories ICCMS™ has defined evidence-based preventive and management strategies to either keep the risk of caries low, or lower the likelihood of caries development. This novel approach provides a link between caries risk status and management of risk.

**Table 2:** ICCMS™ Caries Risk Likelihood Matrix

Patient risk status Based upon the judgment of a dentist or a member of the dental team (including history of restorations and extractions and review of key risk factors)	Current Caries Status at the Patient Level		
	**No active caries lesions**	**Initial-stage active caries lesions**	**Moderate or extensive active caries lesions**

**Low risk**	Low likelihood	Moderate likelihood	Moderate likelihood

**Moderate risk**	Low likelihood	Moderate likelihood	High likelihood

**High risk**	Moderate likelihood	High likelihood	High likelihood

In addition to the Likelihood Matrix of future caries risk, ICCSM™ has developed a matrix to assist dentists in the diagnosis of caries stages. Table 3 defines how the clinical and radiographic staging of caries is integrated to reach to management decisions.

**Table 3:** Diagnostic Stages of Caries based upon Clinical and Radiographic Status

*ICDAS Categories (C)*	*Radiographic Categories (R)*				
	***R0***	***RA_1-2_***	***RA_3_***	***RB***	***RC***

*C_Sound_*	CR_Sound_	CR_Initial_	CR_Initial_	CR_Moderate_	CR_Extensive_

*C_Initial_*	CR_Initial_	CR_Initial_	CR_Initial_*or* CR_Moderate_	CR_Moderate_	CR_Extensive_

*C_Moderate_*	CR_Moderate_	CR_Moderatel_	CR_Moderate_	CR_Moderate_ or CR_Extensive_	Cr_Extensive_

*C_Extensive_*	CR_Extensive_	CR_Extensive_	CR_Extensive_	CR_Extensive_	CR_Extensive_

The ICCMS™ Caries Management Matrix guides the management decisions. The only area where local modifications are possible is the intersection of moderate lesions and RA_3_ (radiolucency reaching the outer one-third of dentin) where the clinical recommendation may be either to manage these lesions non-surgically or surgically, depending on local standards and until further evidence confirm that a more conservative or non-restorative management provide the best outcomes. The levels of management are defined as follows:

M_Initial_: Initial active caries management stage (non-surgical caries management)

M_Moderate_: Moderate active caries management stage (minimally invasive surgical caries management with no or minimal dentin removal)

M_Extensive_: Extensive active caries management stage (invasive surgical caries management with dentin removal)

These management modalities are determined by the diagnostic stage of the caries lesions (Table 3) and activity status.

These two decisions matrices (Tables 2 and 3) represent major advancement in how caries should be managed because they assist dentists to develop comprehensive patient care plans to manage dental caries.

## Keystone #4: ICCMS™ comprehensive patient management plan

After defining an individual likelihood risk status and the management options for caries lesions, a comprehensive patient care plan can be developed that should include the following sections:

**Preventive plan**. The purpose of this plan is to protect sound tooth surfaces from developing new caries lesions. A preventive plan should address both home care and clinical interventions and should be adjusted to the caries risk status of each patient. Based on the best available evidence ICCMS™recommends the following:

## Low risk

Home care: Toothbrushing twice a day with at least 1,000 ppm fluoride dentifrice.

Clinical care: Motivational support to promote healthy dietary and oral hygiene behaviors

## Medium risk

Home care: Same recommendations as for low-risk patients with the addition of either daily (226 ppm F) or weekly (900 ppm F) fluoride rinses, when available. In some countries, higher efficacy fluoride toothpaste such as those containing 1,500 ppm F may be recommended, when available. Using motivational support, dentists or dental team members may work with patients to develop a reduction plan in consumption of sugary drinks, or eating of sugary snacks, and oral hygiene practices.

Clinical care: Dentists may choose from an array of preventive procedures such as sealants, fluoride varnish applied at least twice a year, 2% fluoride gel applied twice a year. In some cases, dentists may consult with other health care providers to reduce the use of recreational drugs or change medication that can reduce salivary flow.

## High risk

In addition to the recommendations for medium risk patients, dentists may prescribe combination therapies that may include chlorhexidine mouthrinse or varnish and fluoride applications; and increase the frequency of fluoride varnish applications to 4 times per year. For home care, dentists may prescribe high concentration preventive dentifrice (5,000 ppm F) for daily use on patients 16 years or older.

## Caries control and restorative care

Caries management is customized to the type of lesion (initial, moderate, and extensive) and activity status. For initial lesions, sealants, fluoride applications, and improvement of oral hygiene practices are recommended.

The ICCMS™TPOP (tooth preserving operative principles) should guide decisions for all restorative care. TPOP is a set of guides that aim to preserve tooth structure, whenever tooth structure is removed surgically. Surgical restorative interventions are only used as a last resort. Where an operative intervention is indicated, ICCMS™ recommends minimal removal of tooth tissues. If an exploratory caries removal is needed, it should be carried out using minimal removal and following the TPOP philosophy. Additionally, in management of initial lesions, tooth separation may be helpful to determine the presence of cavitation. Similar strategies should be used for managing caries lesions in primary teeth. However, if isolation of a primary tooth surface is not feasible prior to placing a resin-based sealant, a glass ionomer sealant is recommended. For root caries, the use of preventive strategies is highly recommended for non-cavitated and even some cavitated lesions.

Minimal removal of enamel and dentin must be followed at all steps of the operative procedures. Cavity preparations should only be sufficient to allow for appropriate removal of dentin caries and to create a peripheral enamel and dentin seal. The shape and extent of the cavity preparation is dictated only by the spread of the caries lesions and presence of infected dentin. Caries removal from the pulpal aspect of the cavity should be carried out to excavate soft infected dentin and prevent exposure of the pulp. It is acceptable to leave discolored carious dentin pulpally. In deep cavities, where there is a risk of pulpal exposure, stepwise or partial excavation should be carried out. Wherever possible, exposure of the dental pulp should be avoided.

ICCMS™ recommends to either seal or repair defective/carious margins wherever possible. This philosophy also applies to defective or lost fissure sealants, which require maintenance and repair only

**Review, monitoring for caries prevention and management:** ICCMS™ differentiates between monitoring of initial lesions to check their progression status and providing preventive interventions, and reviews of behavioral and oral hygiene change plans as well as consultation with other health care providers. These two recall decisions may be scheduled at the same time but they could only be reviewed separately.

It is ICCMS™ position that prevention is an ongoing and dynamic process that involves engaging patients in reviewing their dietary and oral hygiene behaviors as well as clinical preventive care from the first dental visit. ICCMS™ recommends that review and monitoring visits (conventionally referred to as recalls) should be adjusted based upon age of the patient and risk status. The range could be as low as once three months for a child (less than 18 years) with high likelihood of developing caries to a high of once every 2 years for an adult with low likelihood of developing caries (frequency may be adjusted for other conditions).

## Keystone #5: Outcomes of caries management using ICCMS™

Comprehensive patient care plans by design should focus on achieving health outcomes for patients. They should be value-focused and not value-blind plans that should be designed and evaluated to assess the following potential outcomes:

1. Health promotion

a. Number of sound teeth maintained sound

b. Number previously treated teeth maintained free of new disease

2. Disease control

a) Number of initial (active or arrested) caries lesions that remained unchanged or reversed

b) Number of radiographically detected lesions that did not progress or were treated preventively

c) Change in number of teeth with PUFA (pulp, ulceration, fistula and abscess)

3. Patient-centered quality metrics

a) Patients’ satisfaction with their dental health status

b) Patients’ change in care pattern (from episodic to regular care based on risk status)

c) Patients’ caries risk is reduced from high to medium or low status or stabilized if the baseline level was medium or low status

d) Patients’ demonstrate improvement in oral hygiene and dietary practices

e) Reduce cost of overall caries care

## Implementation of ICCMS™

While there have been no studies that have evaluated the ICCMS™system so far, a Global Collaboratory for Caries Management (GCCM) has been formed at King's College London, to initiate comparative studies of the proposed systems and evaluate the process and outcomes of its implementation. There have been several short term and less comprehensive studies in the past of novel management methods of dental caries that preserve tooth structure. Mertz-Fairhurst et al. [80,81] have demonstrated that a conservative enamel and dentin removal and sealing-in of caries can save tooth structure and have favorable outcomes. In addition to the scientific evidence that supports the different interventions proposed in this paper, additional evidence indicates that remineralization of caries lesions can take place in enamel and dentin [82]. An early childhood caries management approach that focuses on home care, prevention, and restorative care resulted in positive outcomes [83]. Sealants and resin infiltration have shown potential of slowing the progression or reversing non-cavitated caries lesions [84-86].

In practice, implementation of the ICCMS™ will require introducing decision tools and education programs to increase the comfort level among dentists that the proposed system is pragmatic, practical, and easy to implement. ICCMS™ treats caries as a disease and not as a lesion [87], is value-focused on tooth preservation because it aims to promote health and assess health outcomes; and it is based on the best current evidence.

The ICCMS model has not yet been fully implemented by dentists from different countries. It represents a departure from the norm on how caries is managed and, as a result, its implementation will require significant changes in behaviors of practitioners. Like all proposed new models in healthcare, the ICCMS system should be modified during the implementation phase to ensure that an objective, simple, and an effective caries management pathway emerges to prevent, control and restore dental caries.

## Authors' contributions

Dr. AII has written the ICCMS manual and this paper with Dr. MT, Dr. NBP co-Chair the International Caries Detection and Assessment System (ICDAS) and he helped to organize and contribute to the workshop that defined the system described in this.

## Supplementary Material

Additional File 1 Definitions of the International Caries Detection and Assessment System (ICDAS™) Codes. Click here for file

Additional File 2 Click here for file

## Declaration

The majority of the cost of writing this paper was paid for by the salaries received by the authors from their respective universities. Colgate Palmolive Inc. partially contributed to organizing the workshop where this paper was presented.

This article has been published as part of *BMC Health Services Research* Volume 15 Supplement 1, 2015: Proceedings of Prevention in practice - making it happen. The full contents of the supplement are available online at http://www.biomedcentral.com/bmchealthservres/supplements/15/S1. Publication charges for this supplement were funded by Colgate Palmolive.
