# Supplementary material for: The International Caries Classification and Management System (ICCMS™) An Example of a Caries Management Pathway
Source: BMC Oral Health. 2015 Sep 15;15(Suppl 1):S9. doi: 10.1186/1472-6831-15-S1-S9 (PMC4580843; doi:10.1186/1472-6831-15-S1-S9)
Supplement: Additional File 1 — Definitions of the International Caries Detection and Assessment System (ICDAS™) Codes. [file 1472-6831-15-S1-S9-S1.docx]

Definitions of the International Caries Detection and Assessment System (ICDAS^TM^) Codes.

**Sound tooth surface: Code 0**

There should be no evidence of caries (either no or questionable change in enamel translucency after prolonged air drying (suggested drying time 5 seconds)). Surfaces with developmental defects such as enamel hypoplasia; fluorosis; tooth wear (attrition, abrasion and erosion), and extrinsic or intrinsic stains will be recorded as sound. The examiner should also score as sound a surface with multiple stained fissures if such a condition is seen in other pits and fissures, a condition which is consistent with non-carious habits (e.g. frequent tea drinking).

**First visual change in enamel: Code 1**

Code 1: Pits and fissures

When seen wet there is no evidence of any change in color attributable to carious activity, but after prolonged air drying (approximately 5 seconds is suggested to adequately dehydrate a carious lesion in enamel) a carious opacity or discoloration (white or brown lesion) is visible that is not consistent with the clinical appearance of sound enamel

OR

When there is a change of color due to caries which is not consistent with the clinical appearance of sound enamel and is limited to the confines of the pit and fissure area (whether seen wet or dry). The appearance of these carious areas is not consistent with that of stained pits and fissures as defined in code 0.

Code 1: Smooth tooth surfaces

When seen wet there is no evidence of any change in color attributable to carious activity, but after prolonged air drying a carious opacity (white or brown lesion) is visible that is not consistent with the clinical appearance of sound enamel. This will be seen from the buccal or lingual surface

**Distinct visual change in enamel: Code 2**

The tooth must be viewed wet. When wet there is a (a) carious opacity (white spot lesion) and/or (b) brown carious discoloration which is wider than the natural fissure/fossa that is not consistent with the clinical appearance of sound enamel (Note: the lesion must still be visible when dry).

**Localized enamel breakdown due to caries with no visible dentin or underlying shadow: Code 3**

The tooth viewed wet may have a clear carious opacity (white spot lesion) and/or brown carious discoloration which is wider than the natural fissure/fossa that is not consistent with the clinical appearance of sound enamel. Once dried for approximately 5 seconds there is carious loss of tooth structure at the entrance to, or within, the pit or fissure/fossa. This will be seen visually as evidence of demineralization (opaque (white), brown or dark brown walls) at the entrance to or within the fissure or pit, and although the pit or fissure may appear substantially and unnaturally wider than normal, the dentin is NOT visible in the walls or base of the cavity/discontinuity.

If in doubt, or to confirm the visual assessment, the WHO/CPI/PSR probe can be used *gently* *across a tooth surface* to confirm the presence of a cavity apparently confined to the enamel. This is achieved by sliding the ball end along the suspect pit or fissure and a limited discontinuity is detected if the ball drops into the surface of the enamel cavity/discontinuity.

**Underlying dark shadow from dentin with or without localized enamel breakdown: Code 4**

This lesion appears as a shadow of discolored dentin visible through an apparently intact enamel surface which may or may not show signs of localized breakdown (loss of continuity of the surface that is not showing the dentin). The shadow appearance is often seen more easily when the tooth is wet. The darkened area is an intrinsic shadow which may appear as grey, blue or brown in color. The shadow must clearly represent caries that started on the tooth surface being evaluated. If in the opinion of the examiner, the carious lesion started on an adjacent surface and there no evidence of any caries on the surface being scored then the surface should be coded “0”.

**Distinct cavity with visible dentin: Code 5**

Cavitation in opaque or discolored enamel exposing the dentin beneath.

The tooth viewed wet may have darkening of the dentin visible through the enamel. Once dried for 5 seconds there is visual evidence of loss of tooth structure at the entrance to or within the pit or fissure – frank cavitation. There is visual evidence of demineralization (opaque (white), brown or dark brown walls) at the entrance to or within the pit or fissure and in the examiner judgment dentin is exposed.

The WHO/CPI/PSR probe can be used to confirm the presence of a cavity apparently in dentin. This is achieved by sliding the ball end along the suspect pit or fissure and a dentin cavity is detected if the ball enters the opening of the cavity and in the opinion of the examiner the base is in dentin. (In pits or fissures the thickness of the enamel is between 0.5 and 1.0 mm. Note the deep pulpal dentin should not be probed)

**Extensive distinct cavity with visible dentin: Code 6**

Obvious loss of tooth structure, the cavity is both deep and wide and dentin is clearly visible on the walls and at the base. An extensive cavity involves at least half of a tooth surface or possibly reaching the pulp.

*A description of the criteria as they apply to mesial-distal and buccal-lingual tooth surfaces is available in <http://www.dundee.ac.uk/dhsru/news/icdas.htm>
