# Supplementary material for: The International Caries Classification and Management System (ICCMS™) An Example of a Caries Management Pathway
Source: BMC Oral Health. 2015 Sep 15;15(Suppl 1):S9. doi: 10.1186/1472-6831-15-S1-S9 (PMC4580843; doi:10.1186/1472-6831-15-S1-S9)

CONSENT WAS OBTAINED FROM INDIVIDUALS FOR REPRODUCTION OF EACH OF THESE IMAGES. CONSENT WAS GAINED BY VARIOUS MEMERS OF THE ICDAS TEAM

Appendix

ICCMS^{TM} Caries Staging

Photographs and Radiographs

Pits and fissures

Clinically initial caries lesions

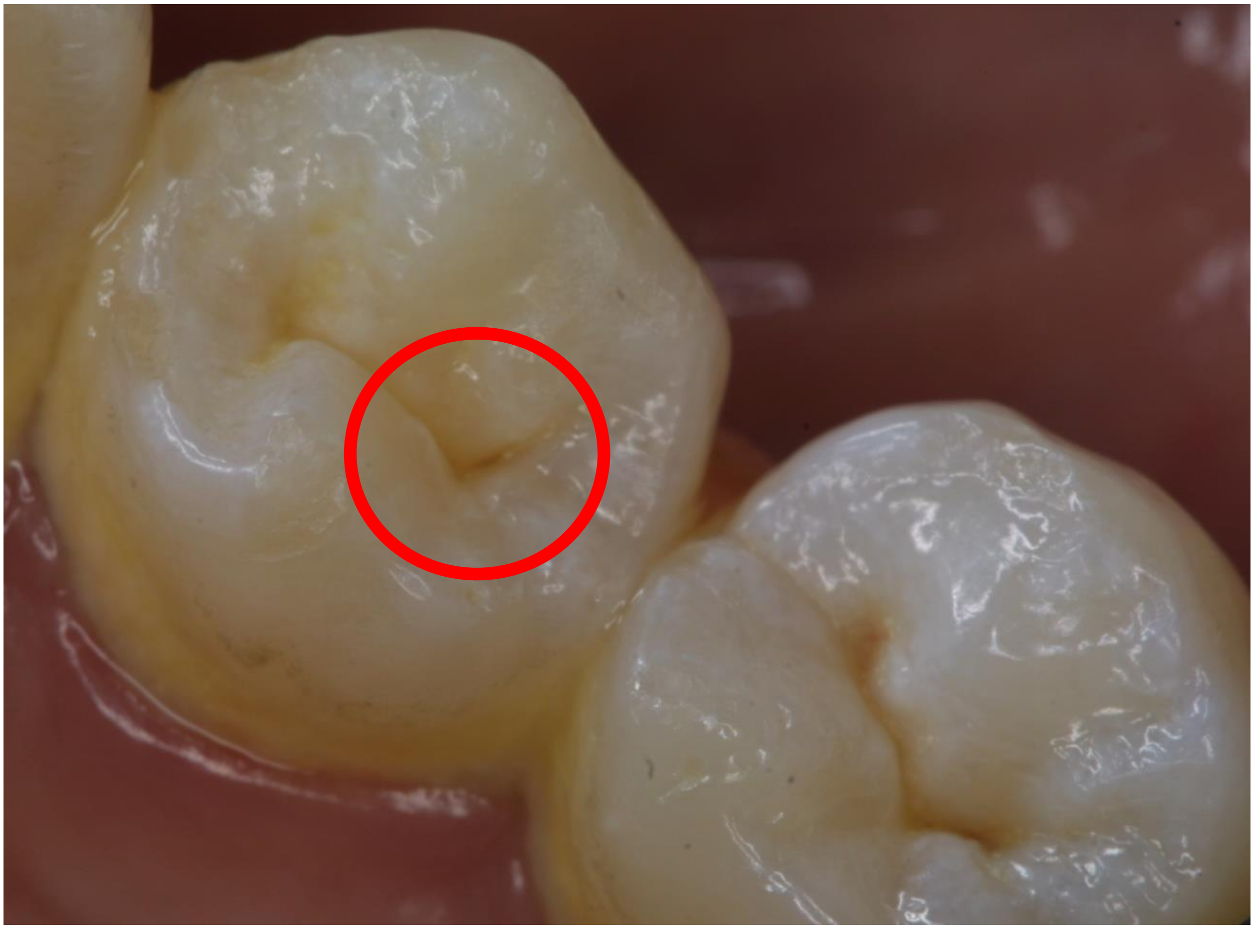

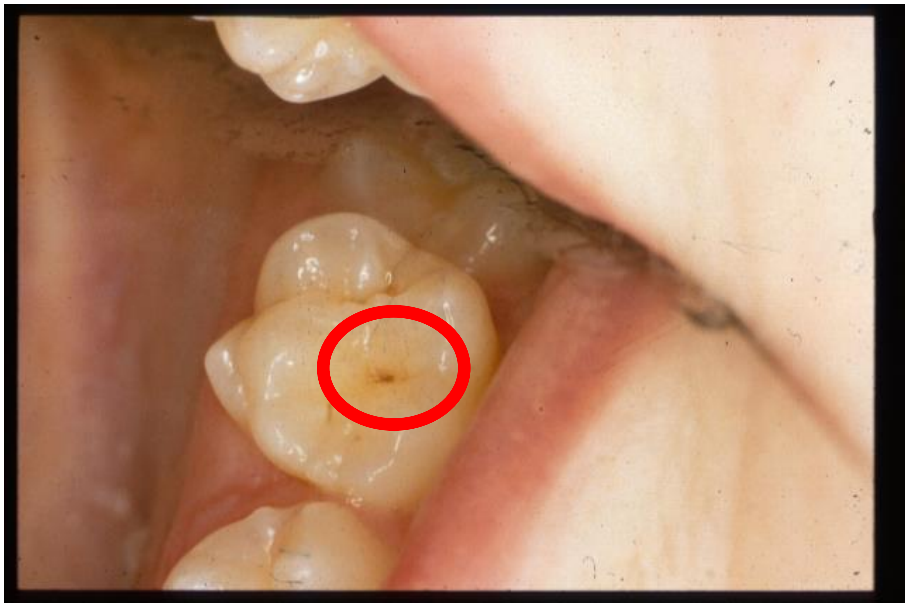

Clinically moderate caries lesions (after examining the radiographs some of the lesions may be classified as extensive)

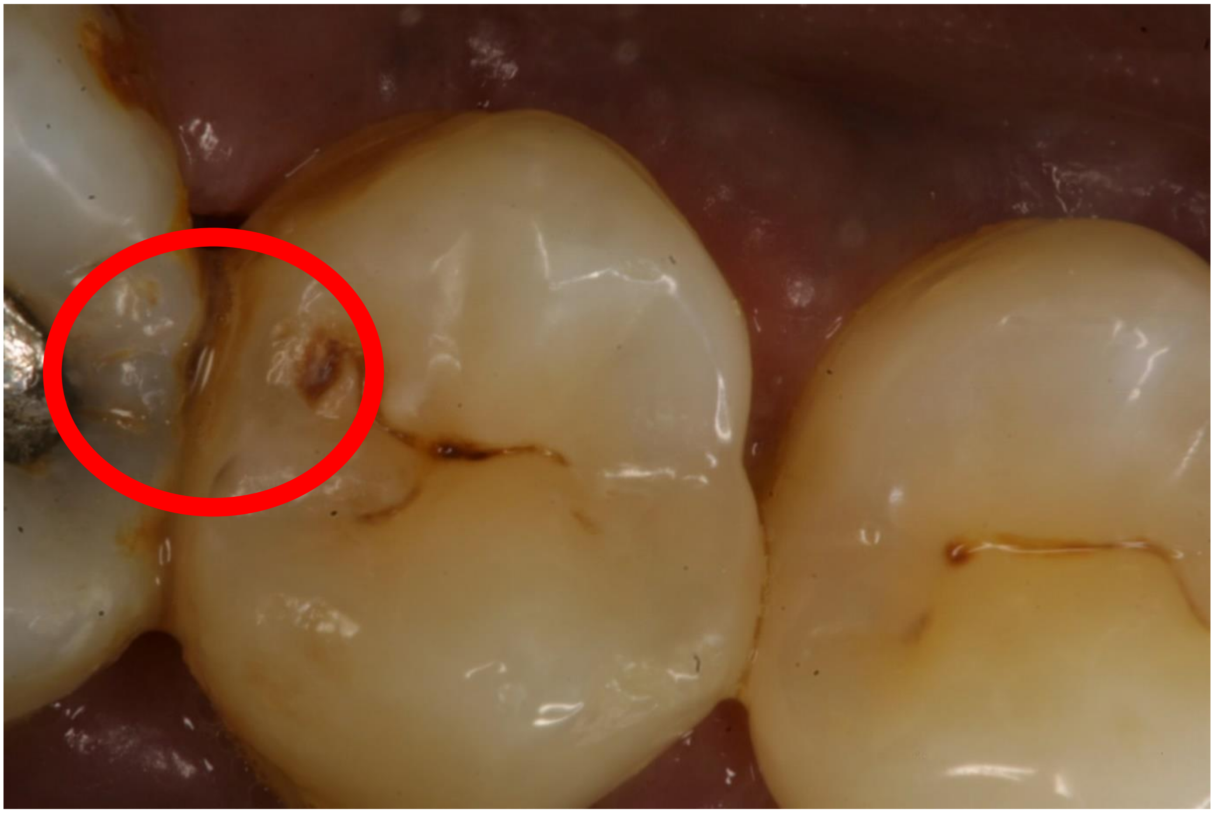

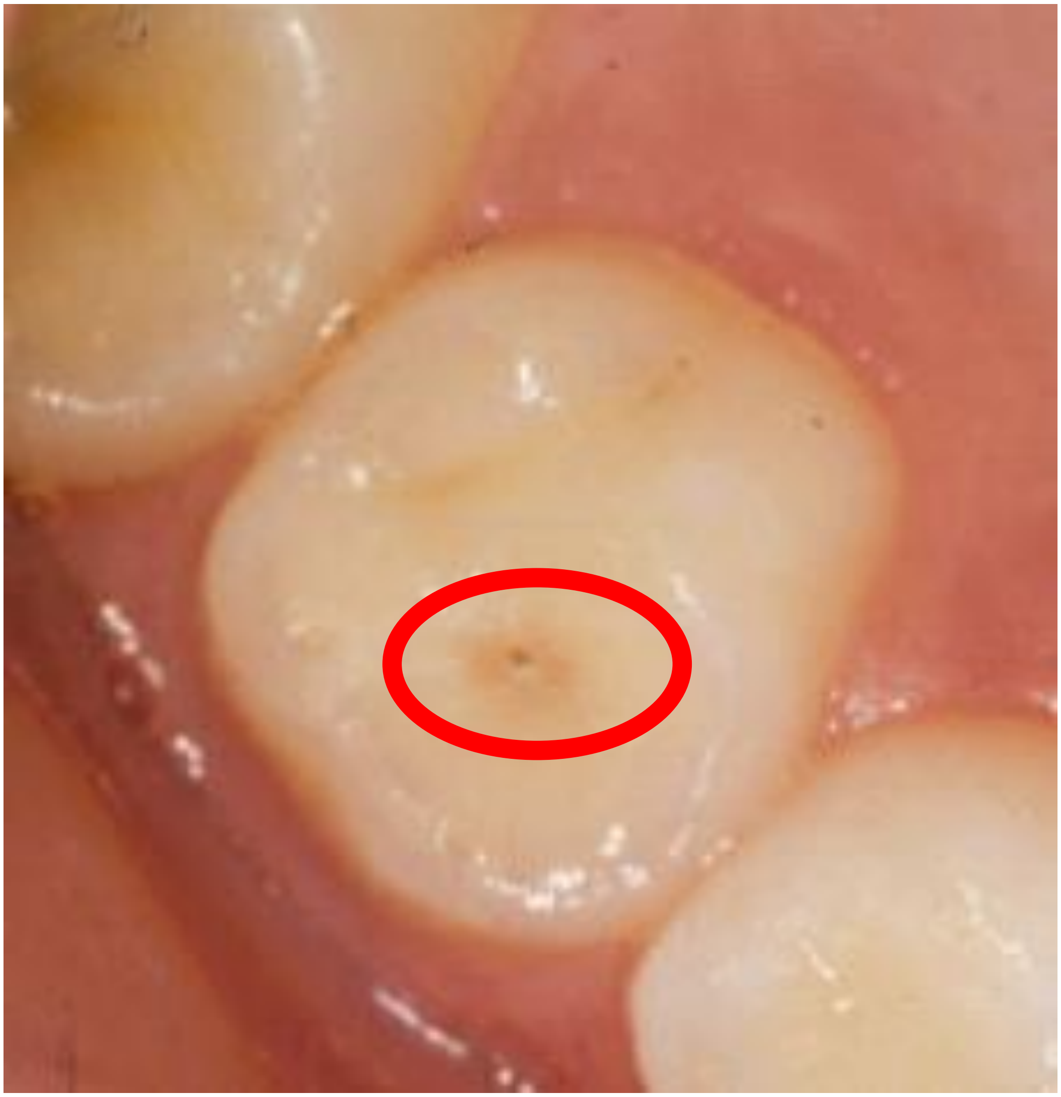

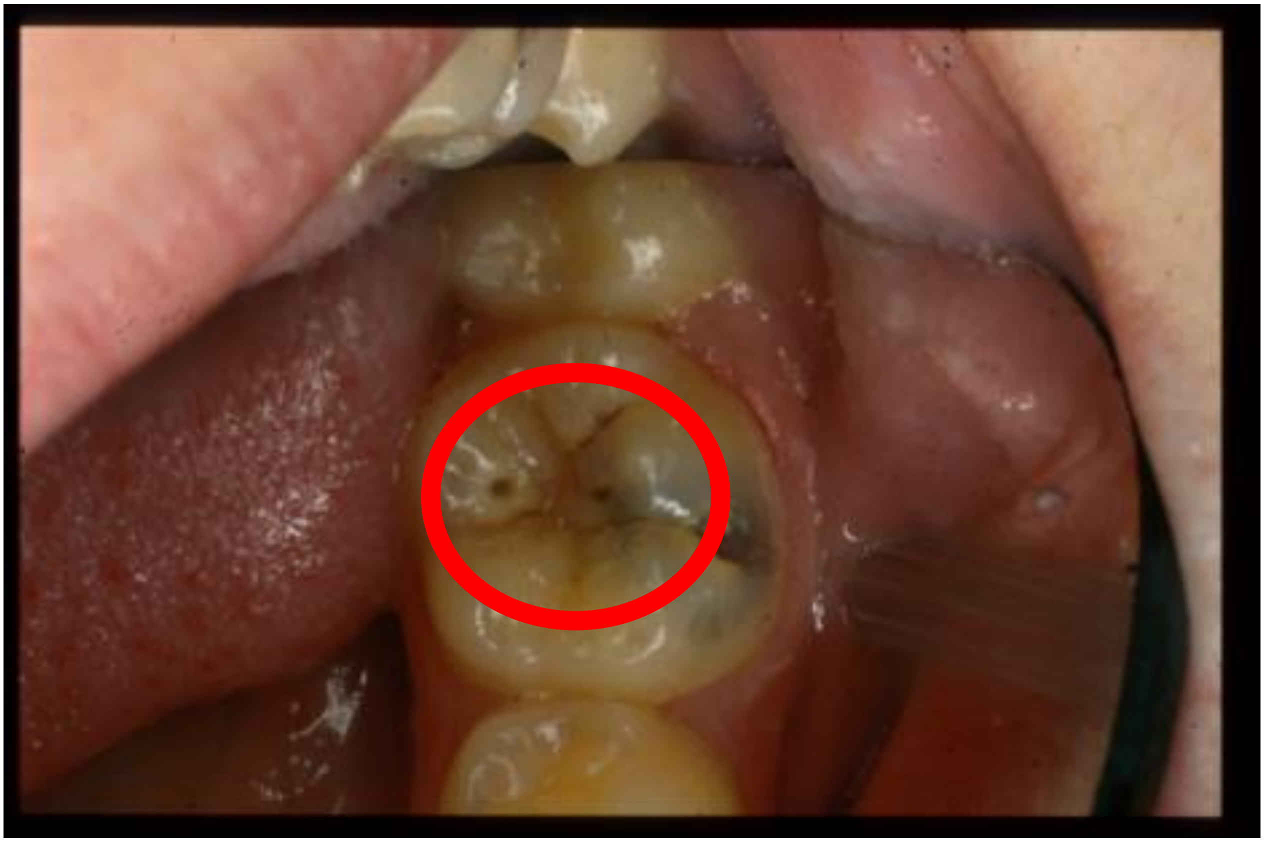

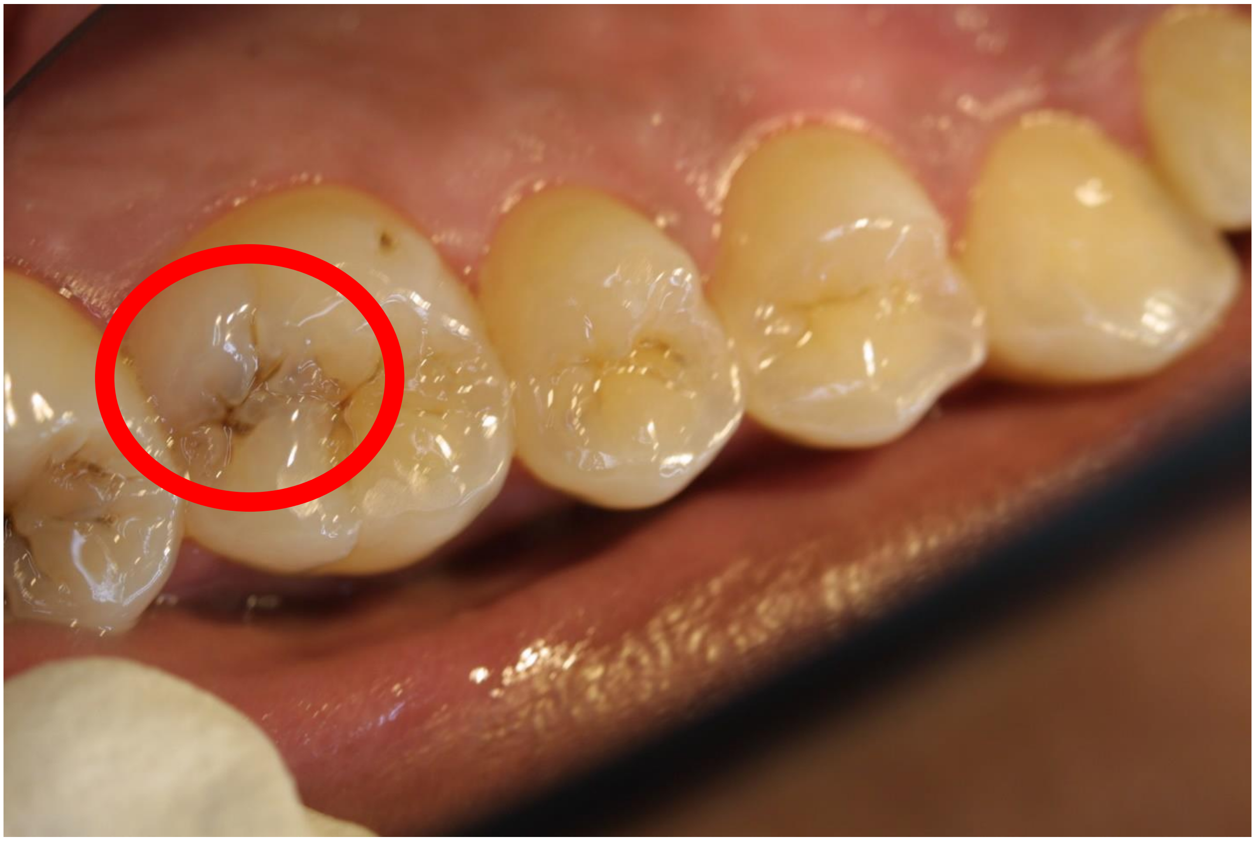

Clinically extensive caries lesions

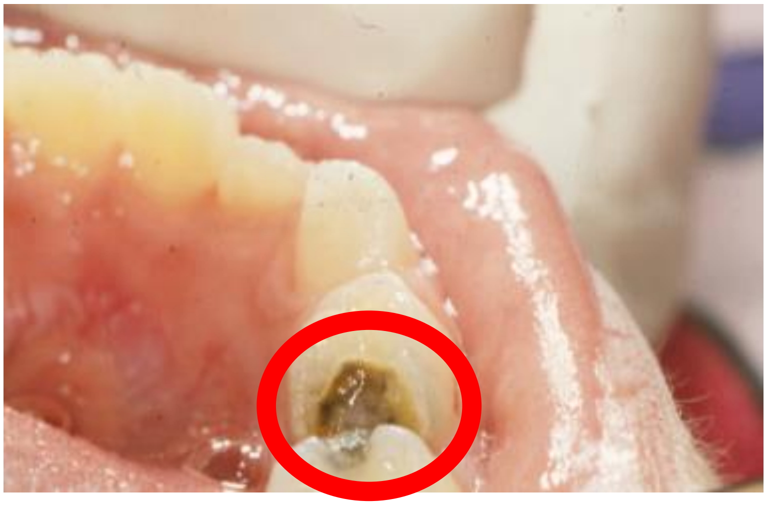

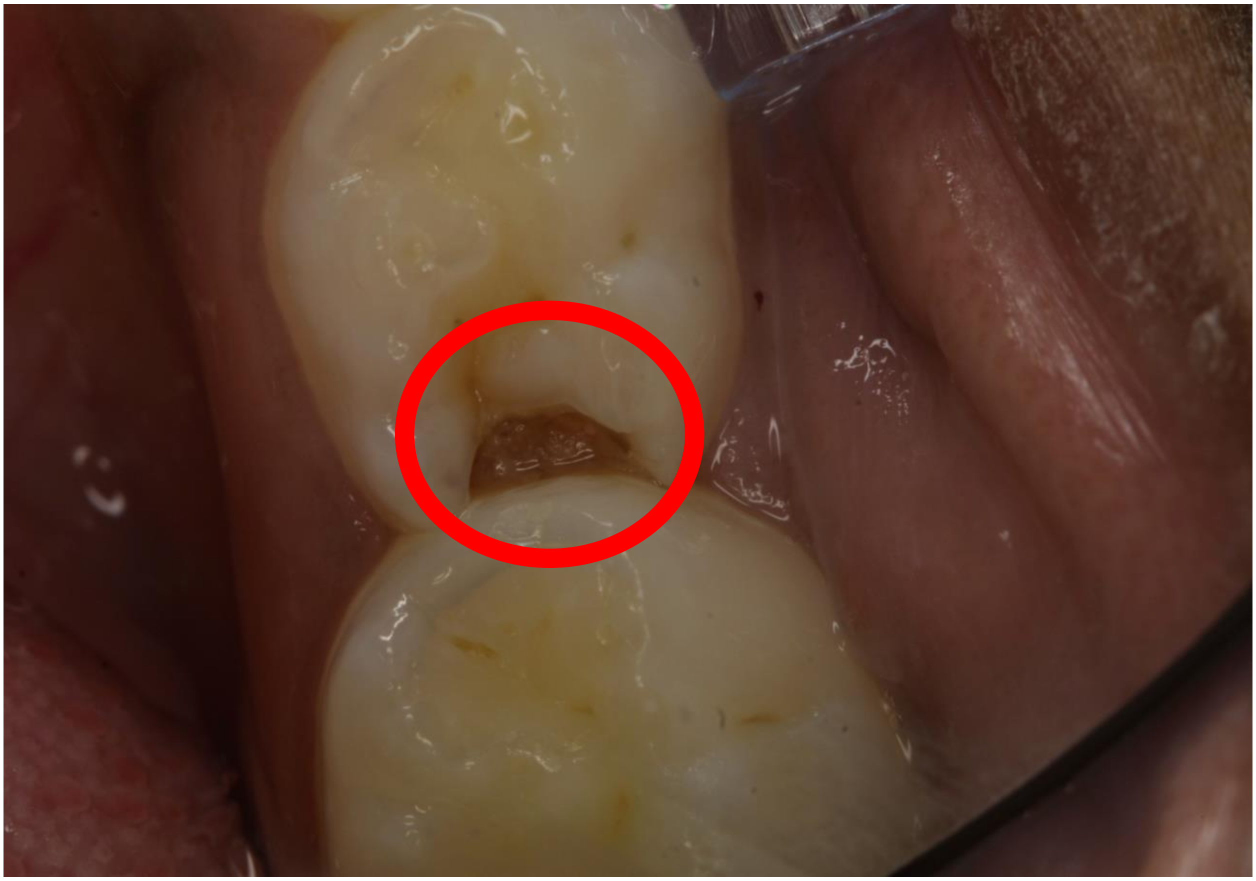

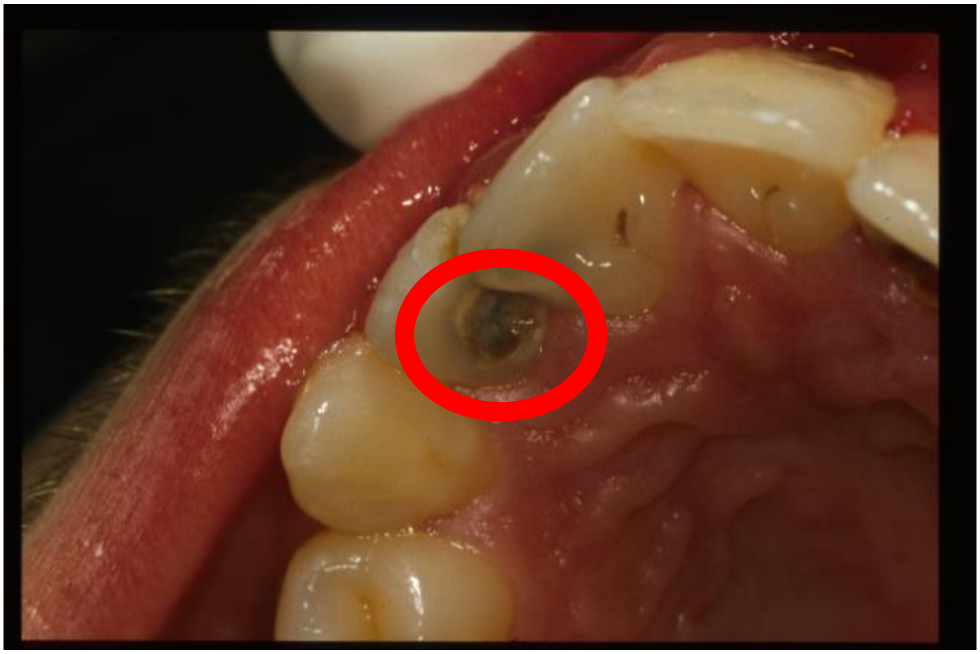

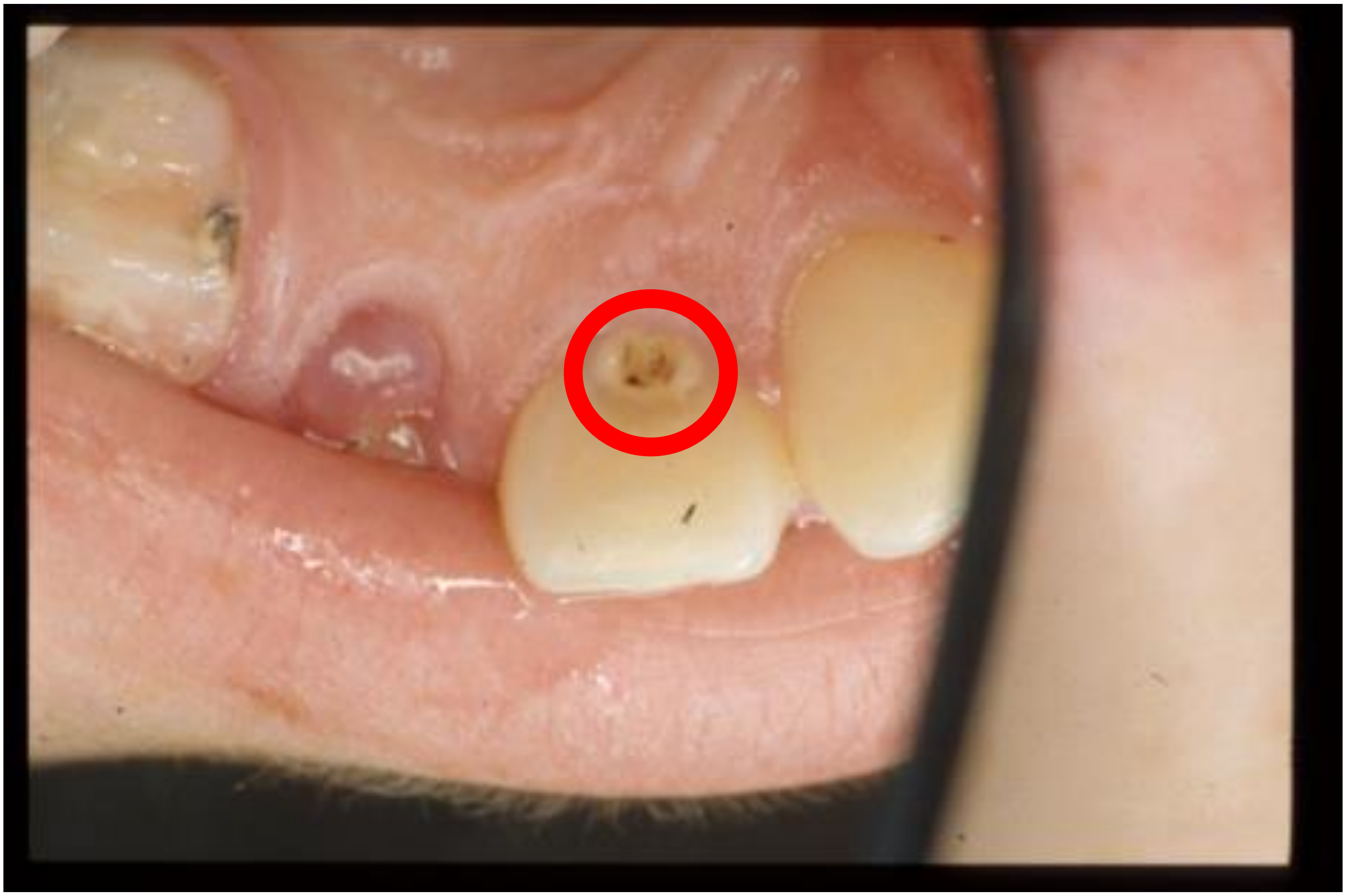

Smooth tooth surfaces

Clinically initial caries lesions

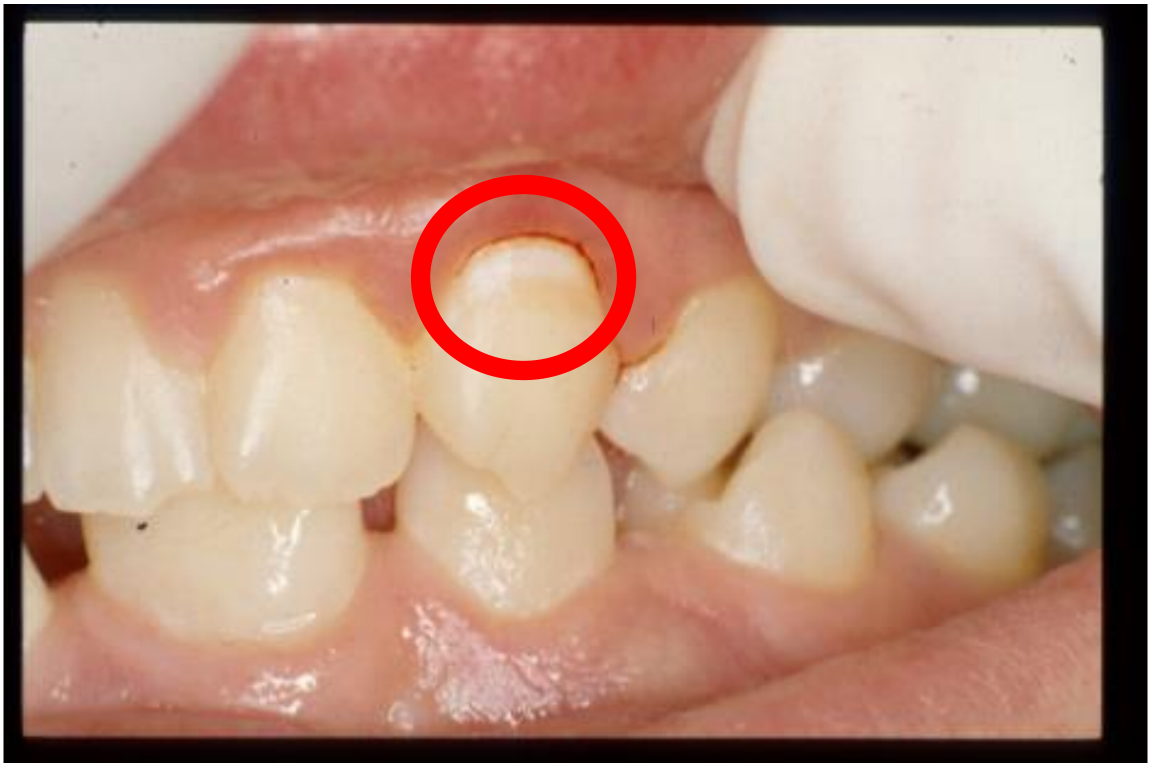

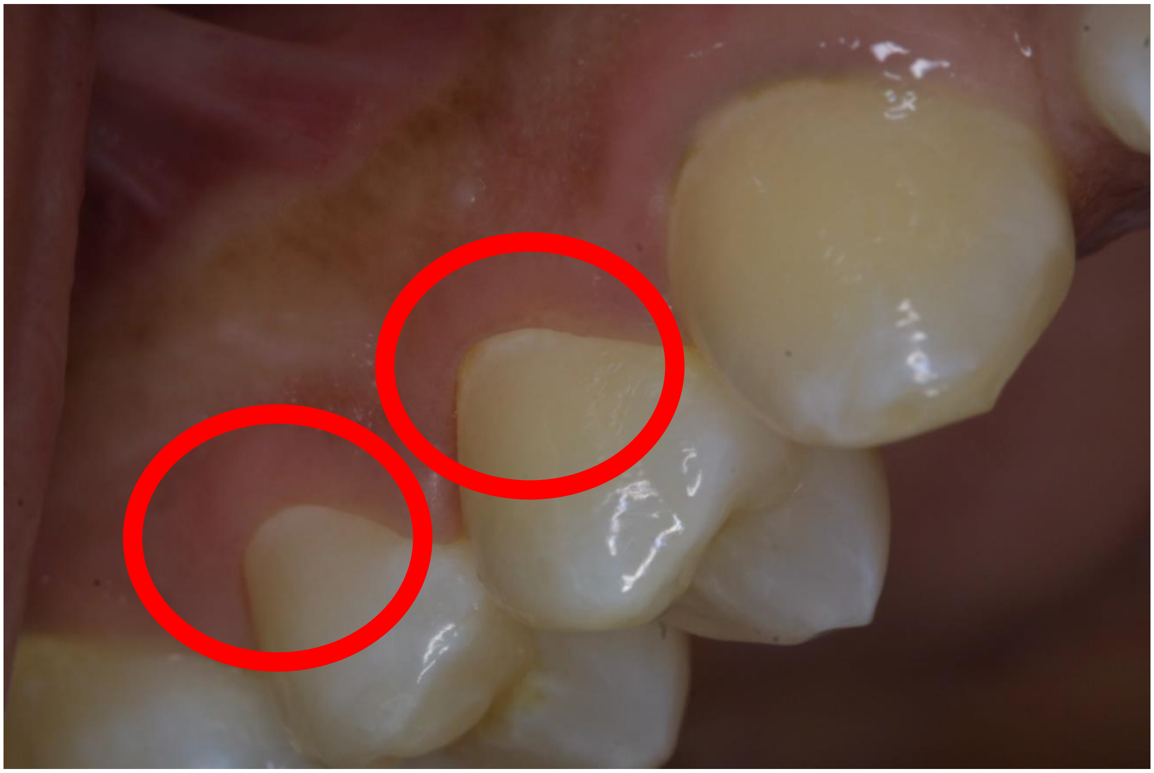

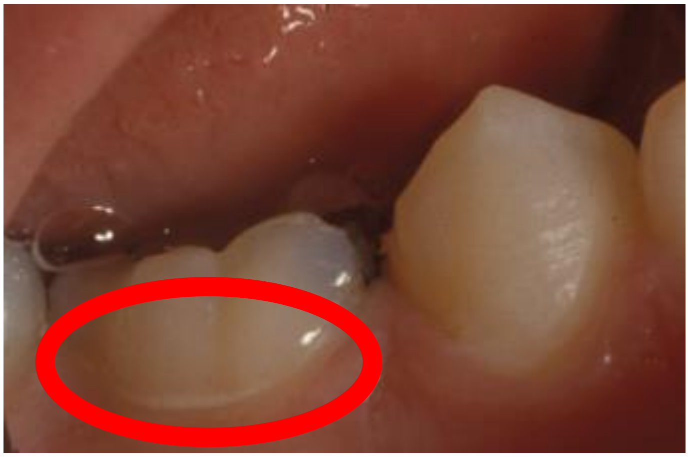

Clinically moderate caries lesions

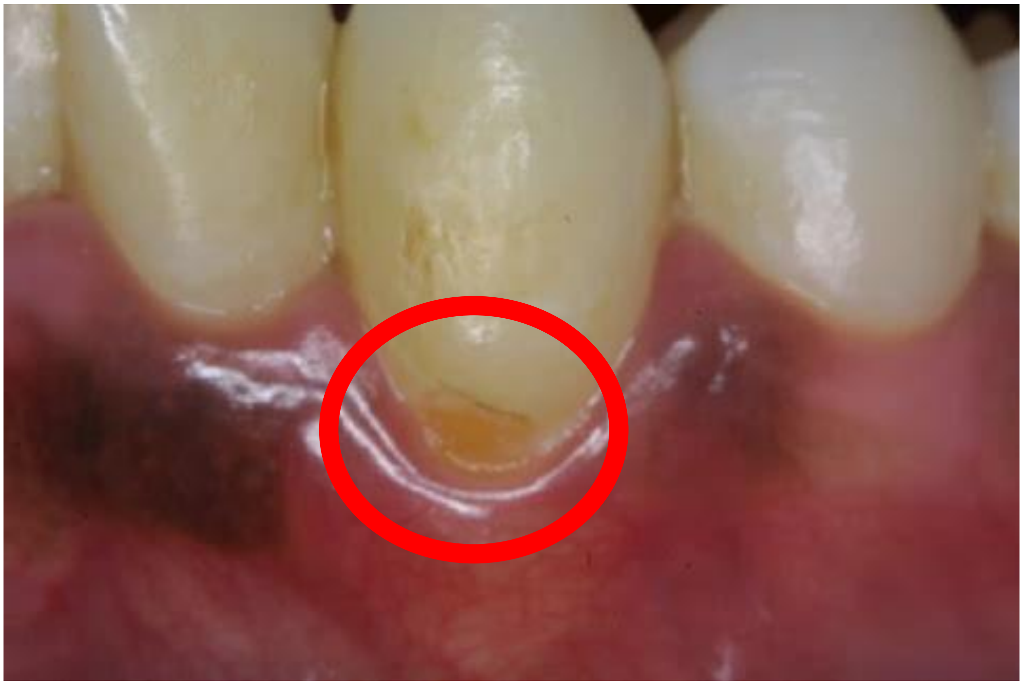

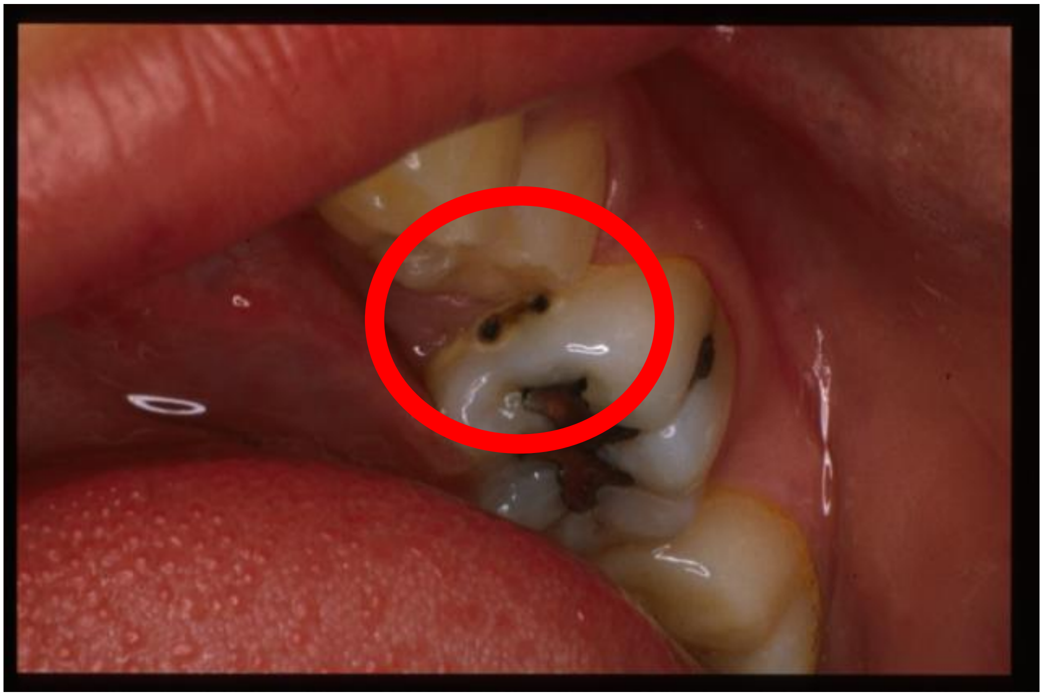

Extensive caries lesions

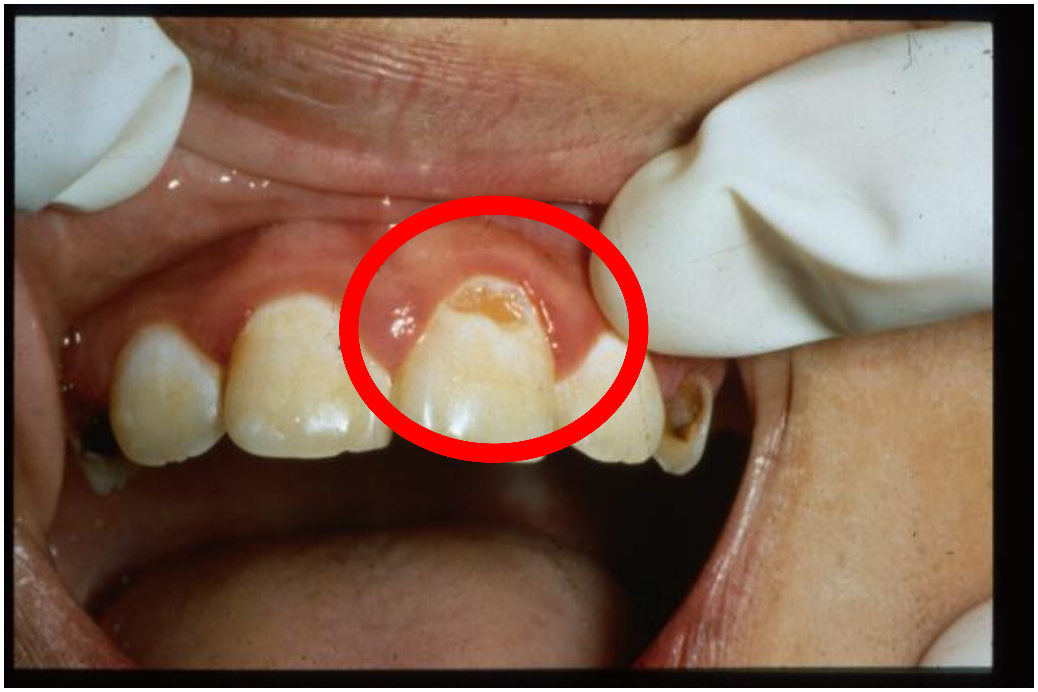

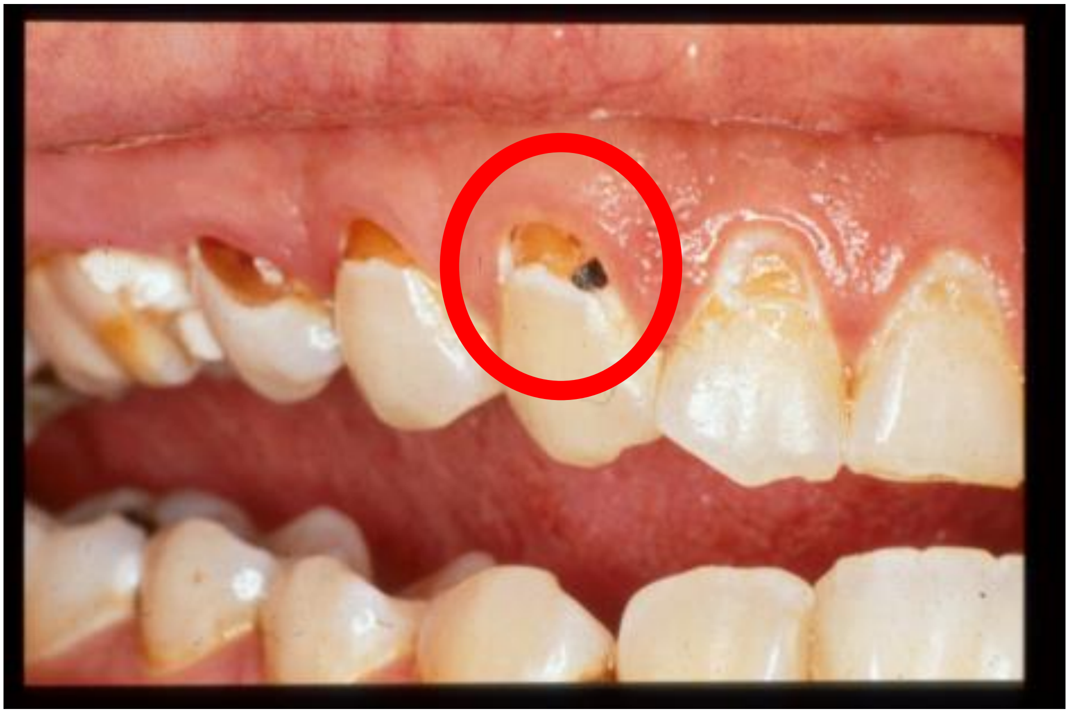

Root Caries

Clinically non-cavitated lesions

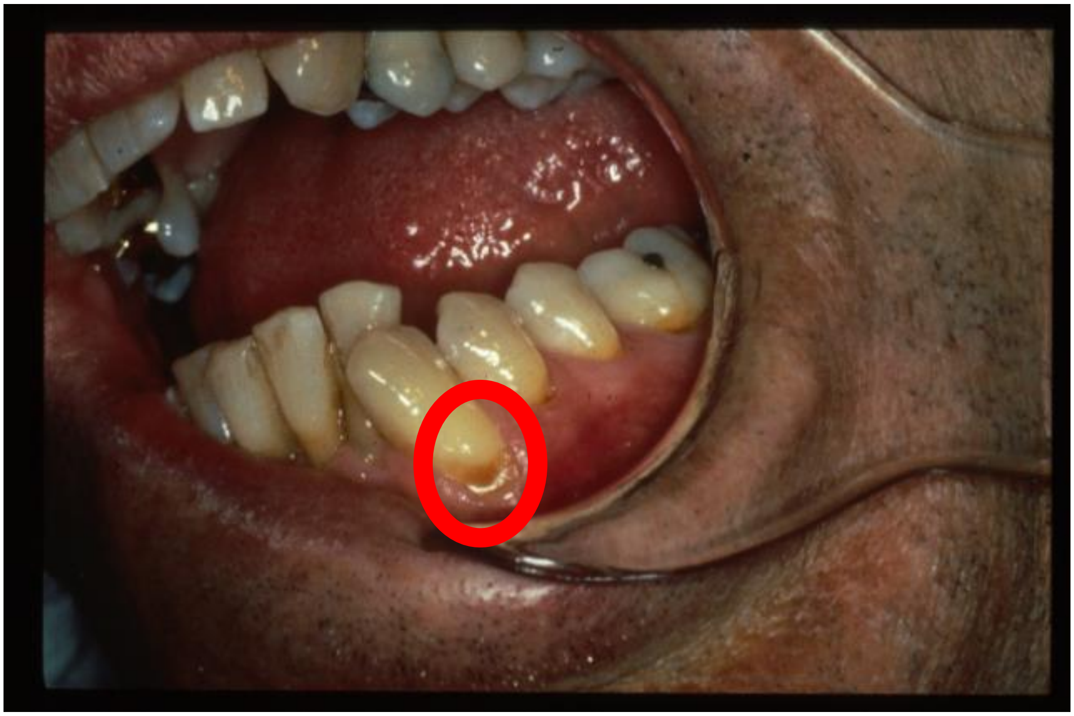

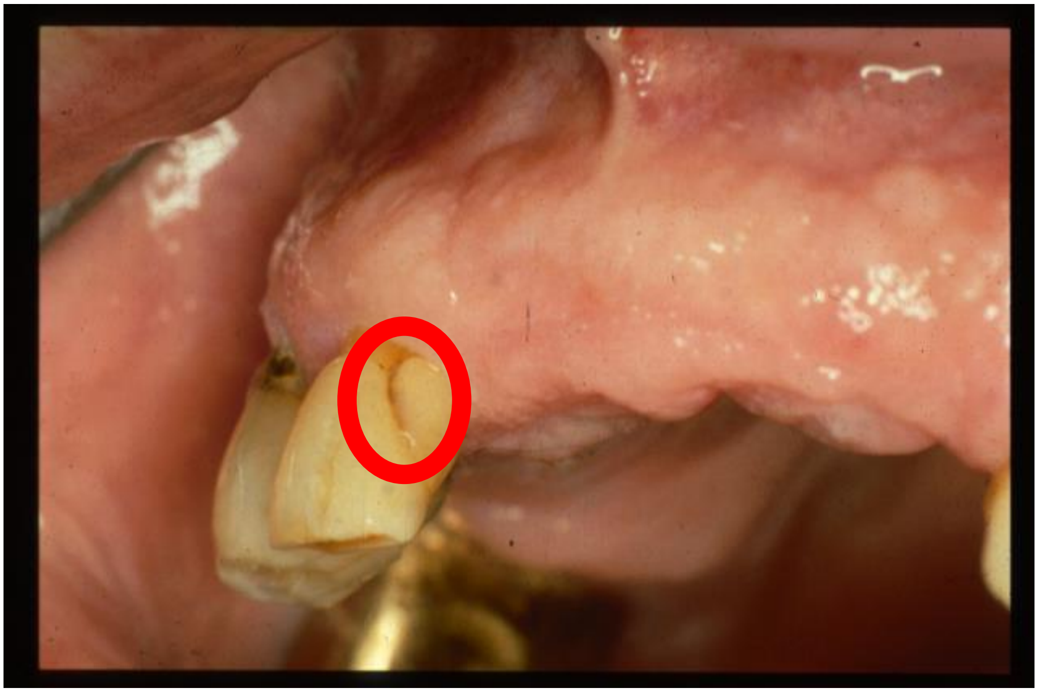

Clinically cavitated lesions

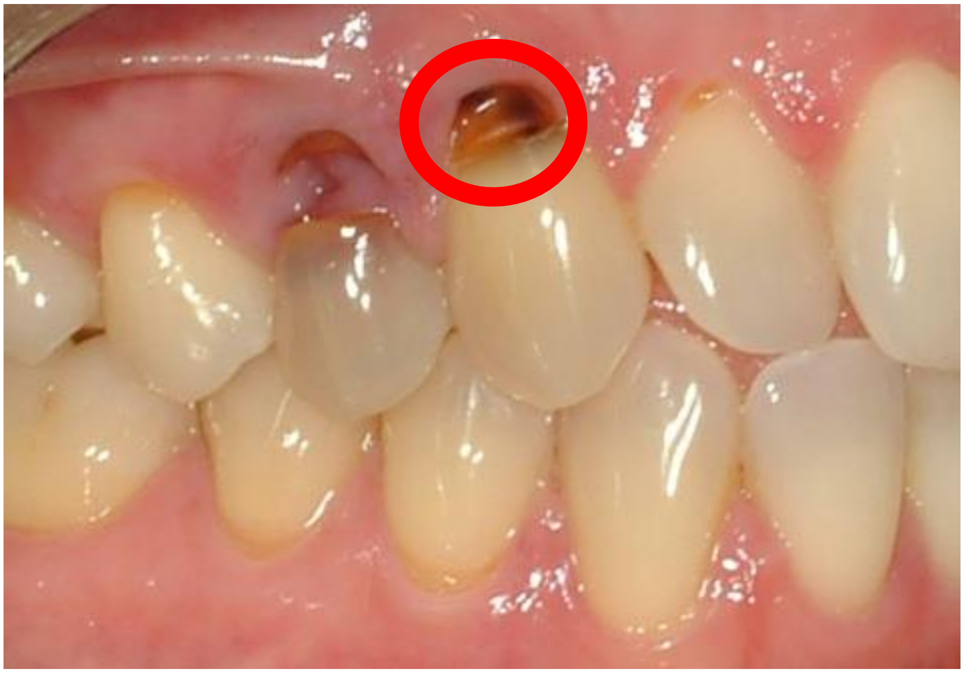

Courtesy of bigdiastema.com

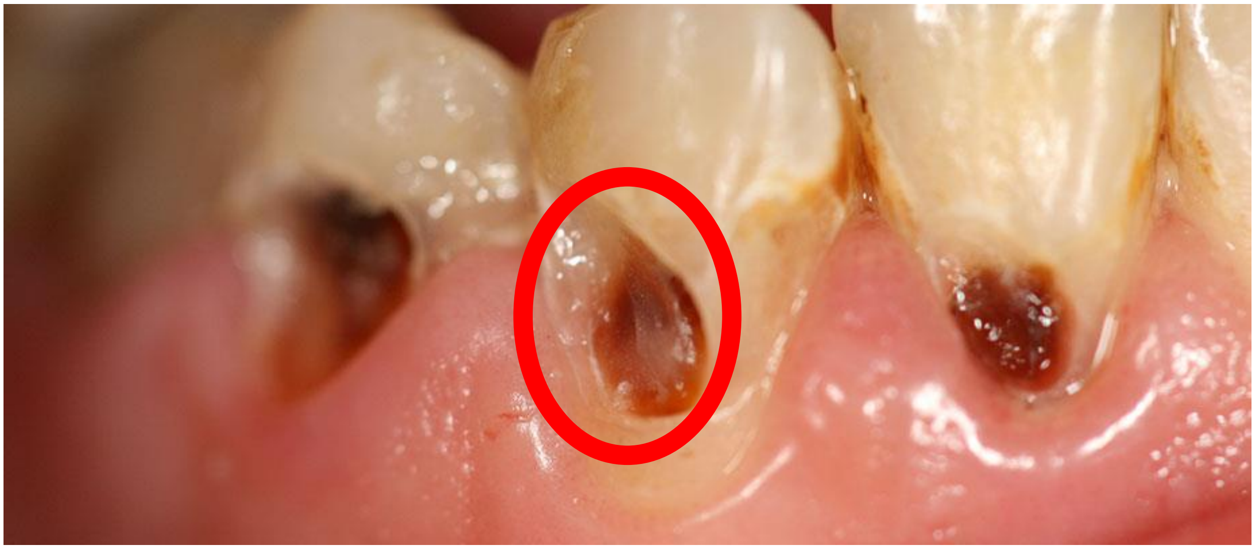

Courtesy of lifecaredental.com.au

Radiographic caries lesions

RA: Initial stages

1= radiolucency in the outer ½ of the enamel

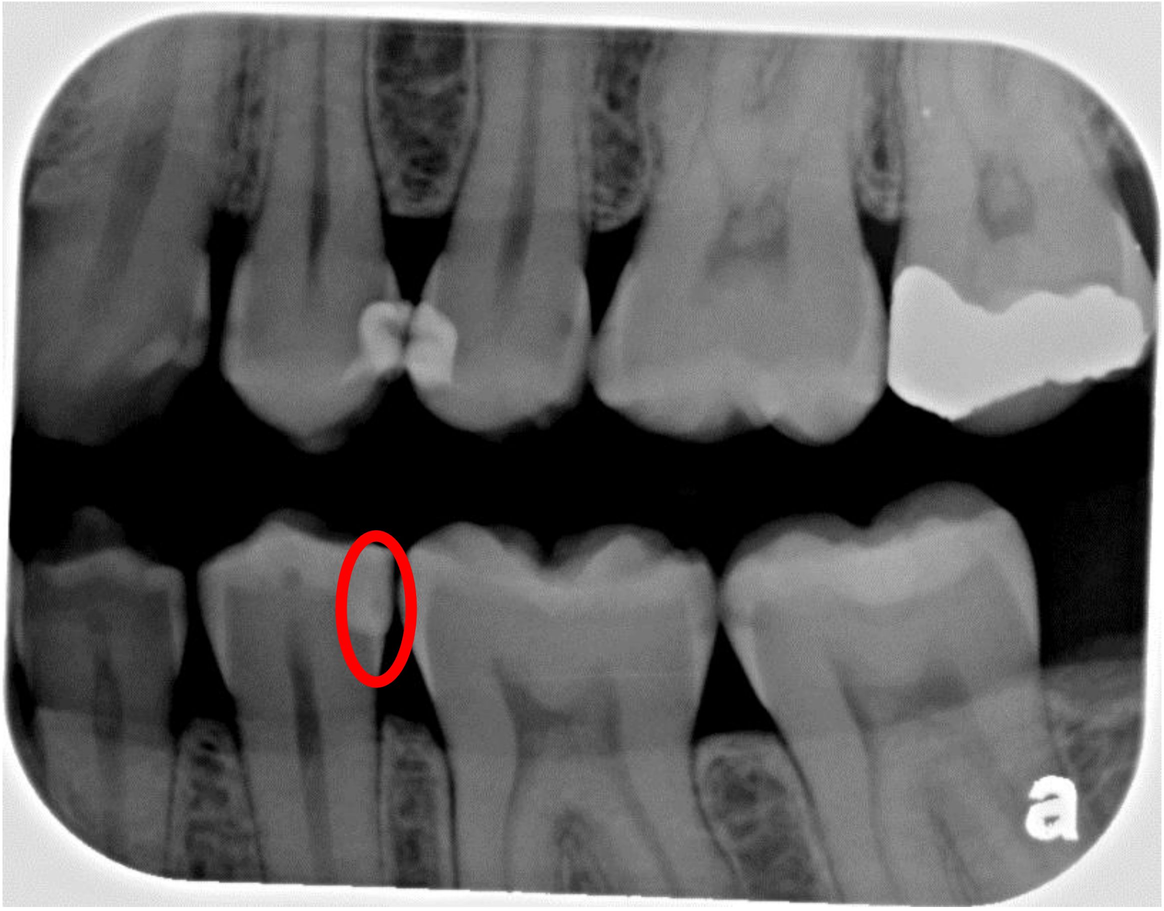

2= radiolucency in the inner ½ of the enamel ± EDJ

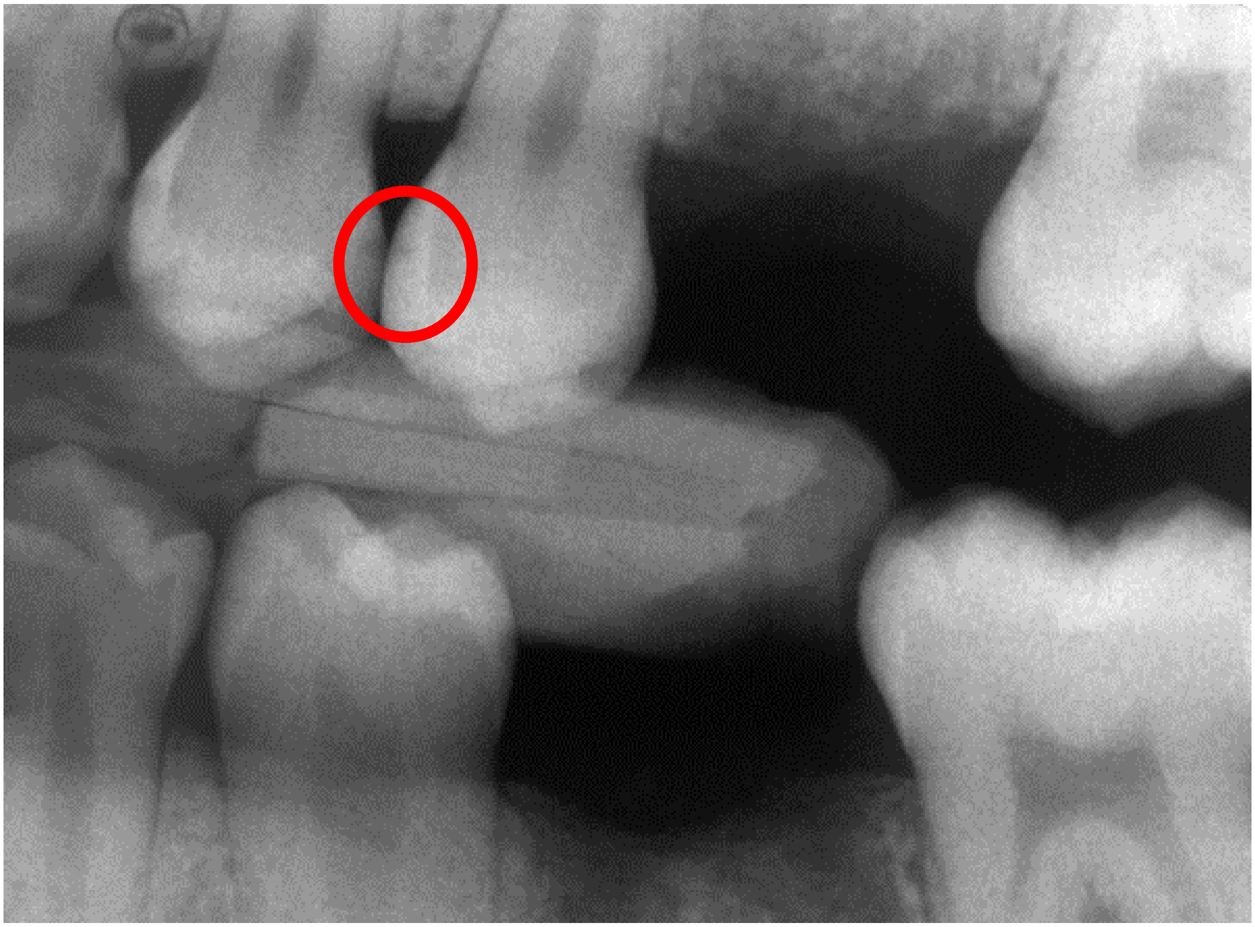

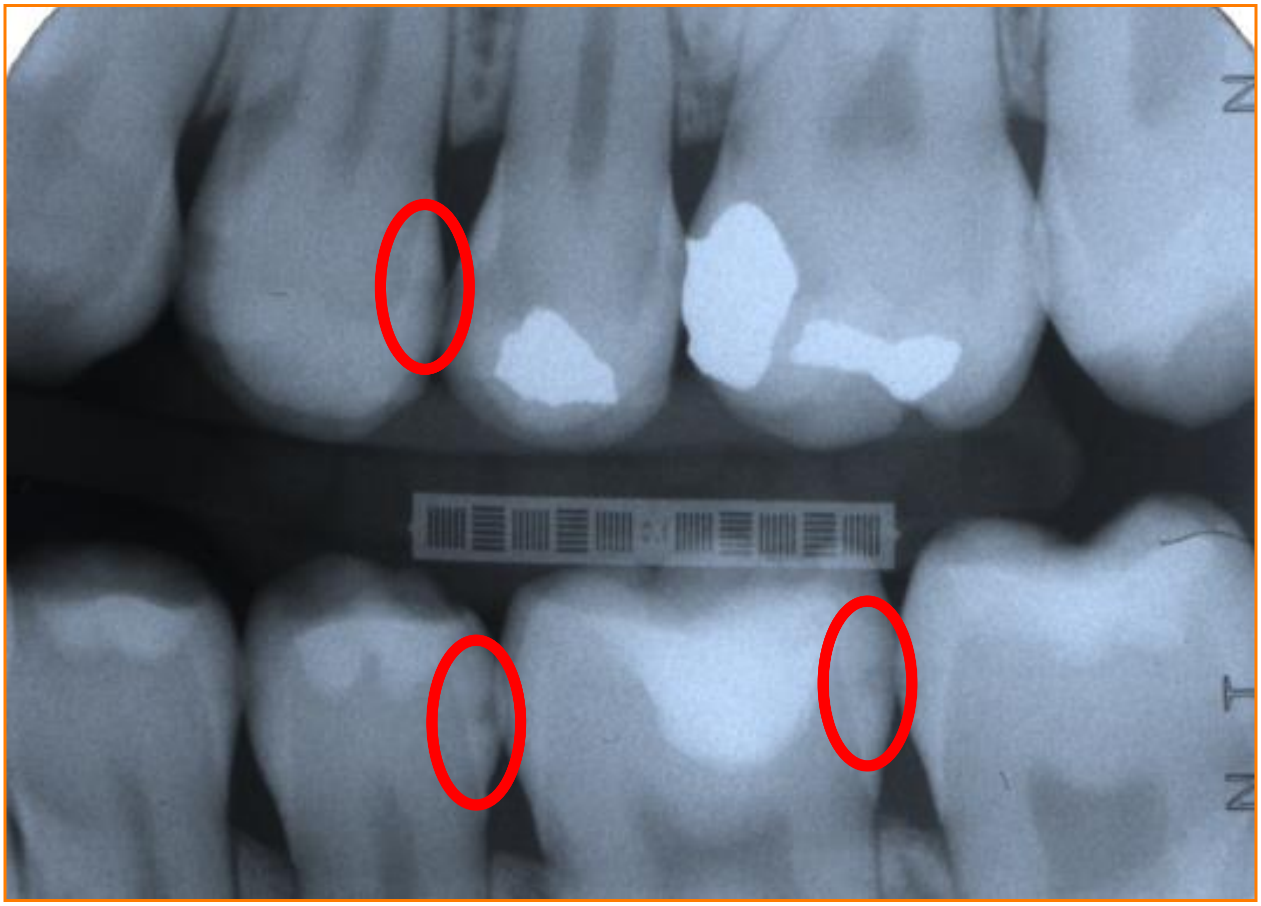

3= radiolucency limited to the outer 1/3 of dentin

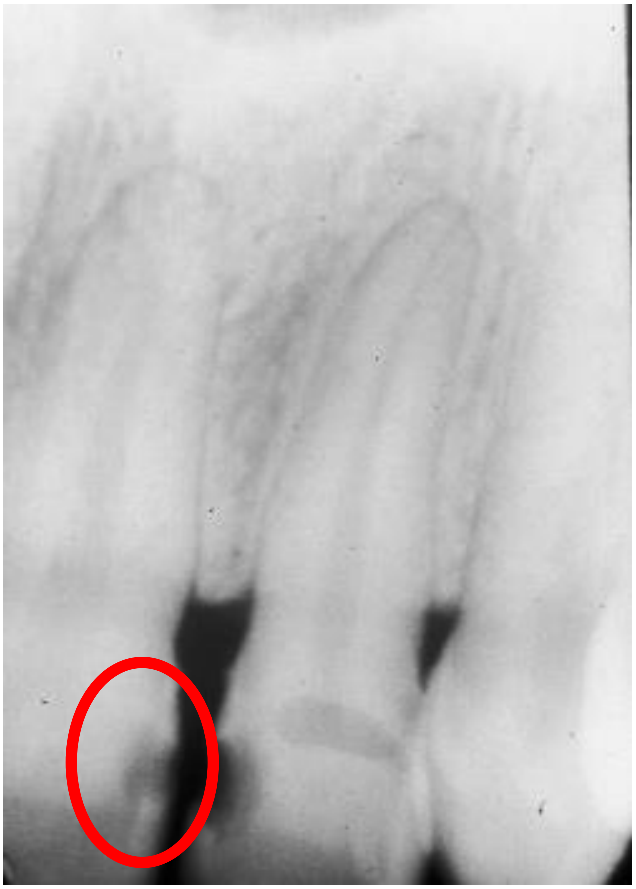

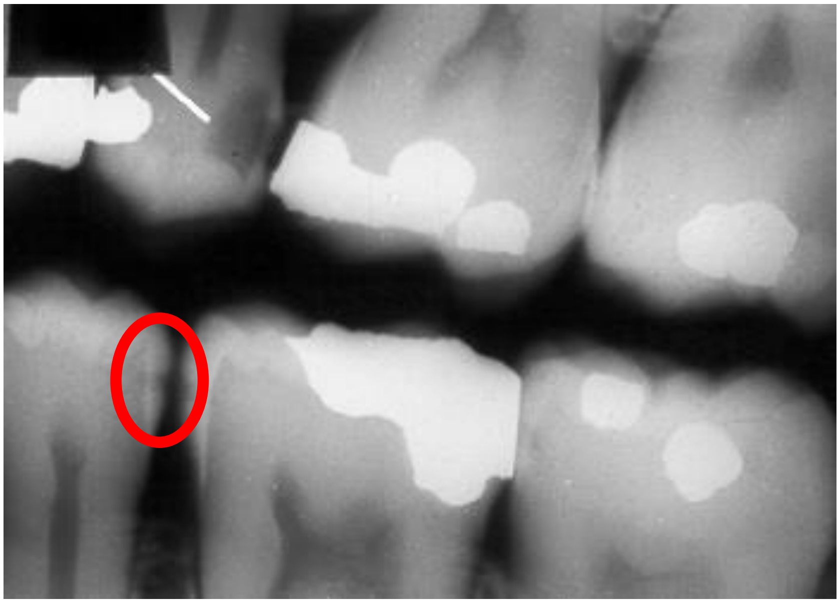

RB: Moderate stages

4= radiolucency reaching the middle 1/3 of dentin

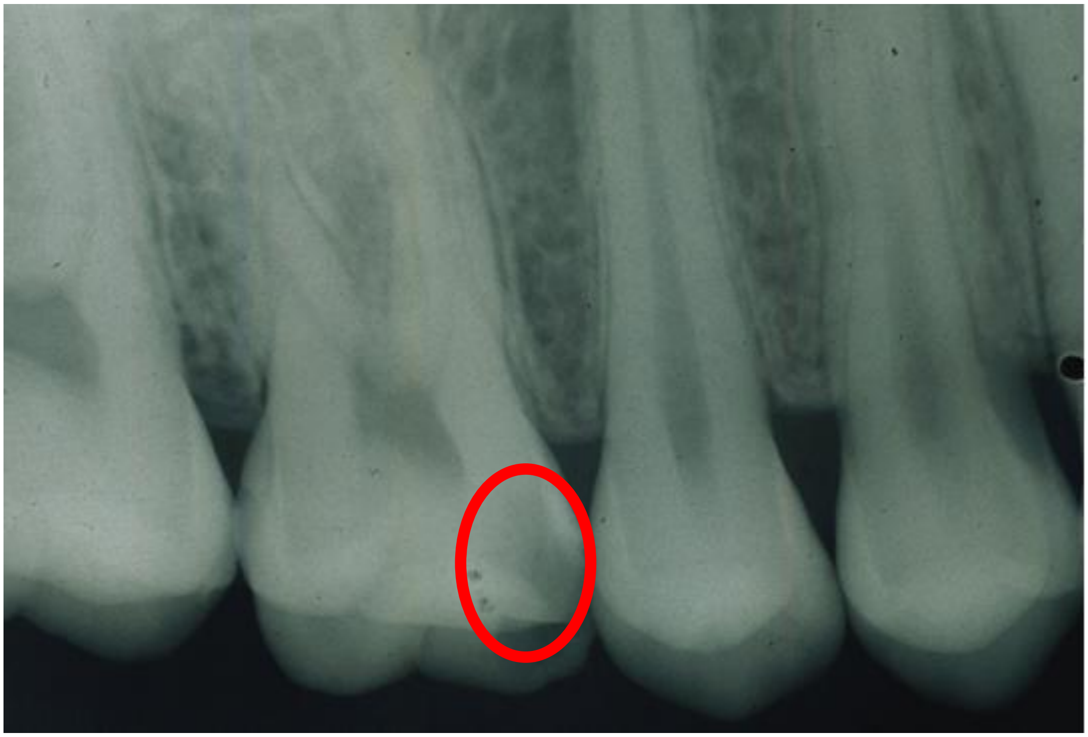

Courtsey of http://dc681.4shared.com/doc/Q-C5tOw2/preview.html

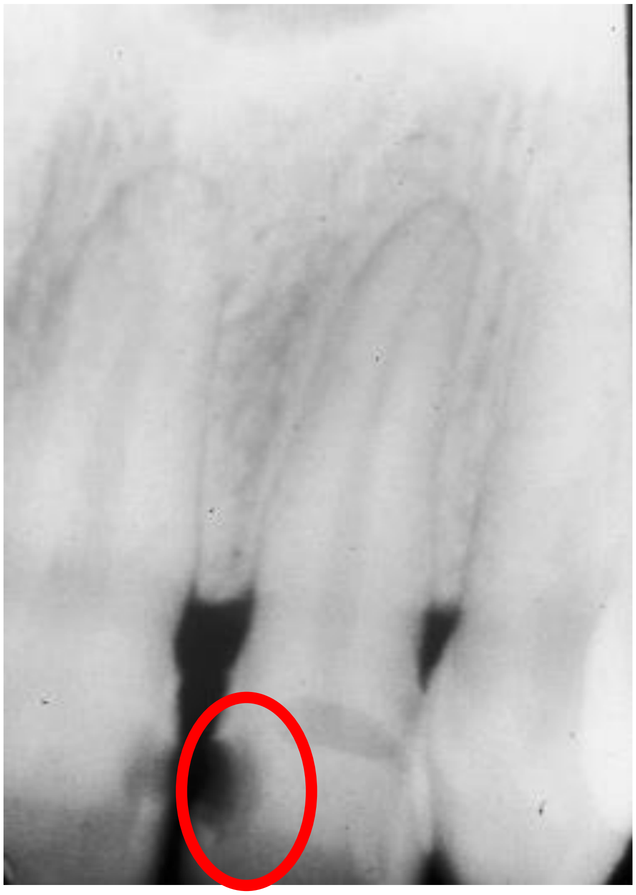

RC: Extensive stages

5= radiolucency in the inner 1/3 of dentin

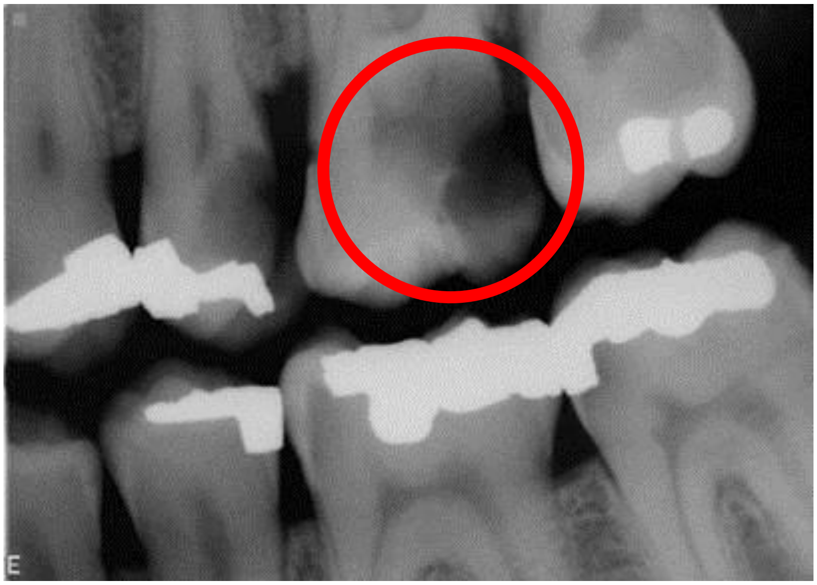

Courtesy of www.hillam.net

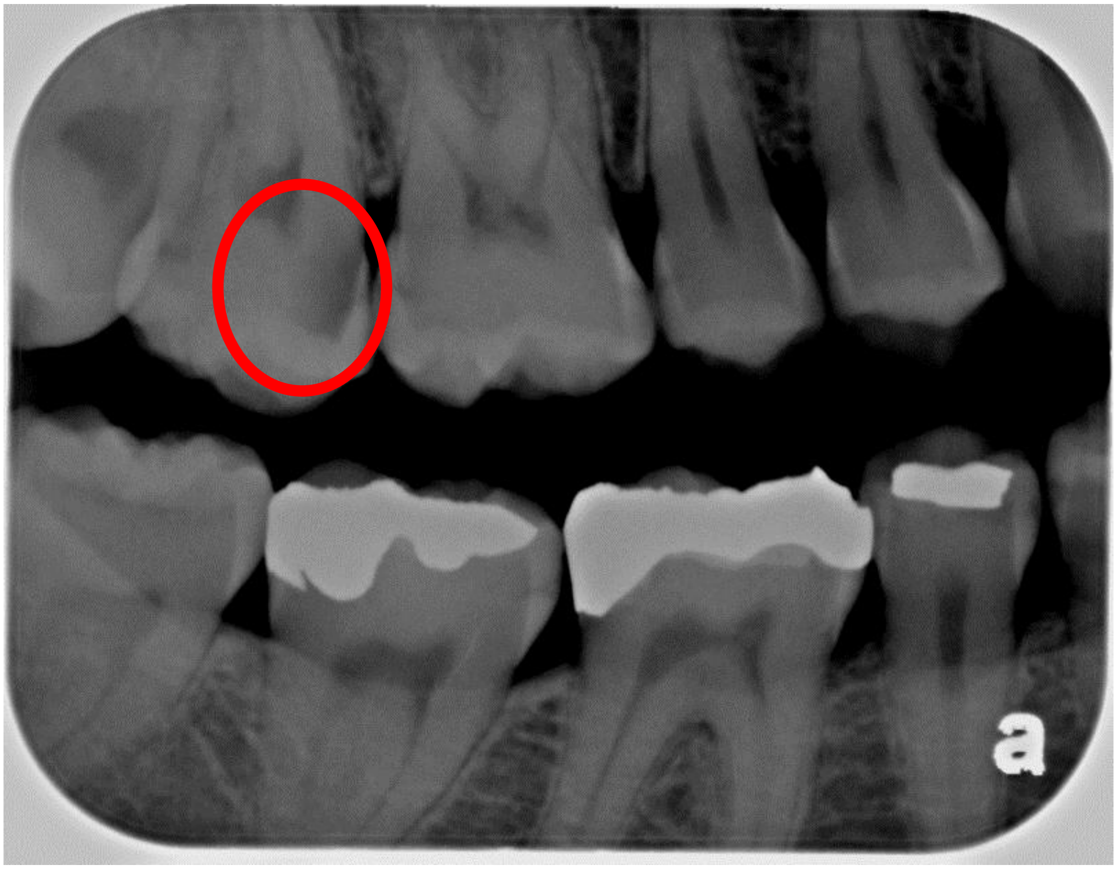
a

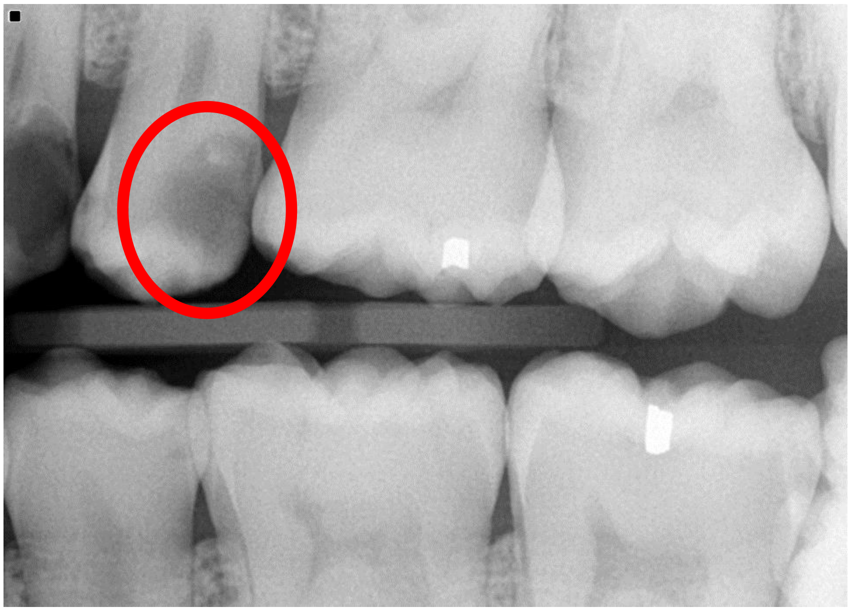

Supplement: Additional File 2 [file 1472-6831-15-S1-S9-S2.pdf]
